# Comparing delivery channels to promote nutrition-sensitive agriculture: A cluster-randomized controlled trial in Bangladesh

**DOI:** 10.1016/j.foodpol.2023.102484

**Published:** 2023-07

**Authors:** Akhter Ahmed, Fiona Coleman, John Hoddinott, Purnima Menon, Aklima Parvin, Audrey Pereira, Agnes Quisumbing, Shalini Roy

**Affiliations:** a International Food Policy Research Institute, Washington, DC, United States; b Cornell University, Ithaca, NY, United States; c University of North Carolina, Chapel Hill, NC, United States

**Keywords:** Extension agents, Community nutrition workers, Nutrition knowledge, Diet quality, Women’s empowerment, Bangladesh

## Abstract

We use a randomized controlled trial in rural Bangladesh to compare two models of delivering nutrition content jointly to husbands and wives: deploying female nutrition workers versus mostly male agriculture extension workers. Both approaches increased nutrition knowledge of men and women, household and individual diet quality, and women’s empowerment. Intervention effects on agriculture and nutrition knowledge, agricultural production diversity, dietary diversity, women’s empowerment, and gender parity do not significantly differ between models where nutrition workers versus agriculture extension workers provide the training. The exception is in an attitudes score, where results indicate same-sex agents may affect scores differently than opposite-sex agents. Our results suggest opposite-sex agents may not necessarily be less effective in providing training. In South Asia, where agricultural extension systems and the pipeline to those systems are male-dominated, training men to deliver nutrition messages may offer a temporary solution to the shortage of female extension workers and offer opportunities to scale and promote nutrition-sensitive agriculture. However, in both models, we find evidence that the presence of mothers-in-law within households modifies the programs’ effectiveness on some nutrition, empowerment, and attitude measures, suggesting that accounting for other influential household members is a potential area for future programming.

## Highlights

• Using an RCT, we compare two models of delivering nutrition content jointly to husbands and wives: deploying female nutrition workers versus mostly male agriculture extension workers.
• Both approaches increased nutrition knowledge of men and women, household and individual diet quality, and women’s empowerment; however, we do not reject the null that their impacts were equal.
• Our results suggest opposite-sex agents may not necessarily be less effective in providing training. In South Asia, where agricultural extension systems are male-dominated, training men to deliver nutrition messages may offer a temporary solution to the shortage of female extension workers and offer opportunities to scale and promote nutrition-sensitive agriculture.
• However, in both models, we find evidence that the presence of mothers-in-law within households attenuates the programs’ effectiveness on some nutrition, empowerment, and attitude measures.

## 1 Introduction

Making agriculture “nutrition-sensitive” is increasingly recognized as a strategy to improve diets and nutrition in developing countries at scale (Ruel et al., 2018). This approach implicitly assumes collaboration between the agriculture and nutrition sectors, yet little evidence-based guidance exists on how the sectors should collaborate. Because cross-sectoral programs are complex to design and coordinate, Ruel and Alderman (2013) ask whether different sectors should focus on “integration” (joint planning, implementation, monitoring, and assessment) or effective “co-location” (implementing programs managed by different sectors to reach and saturate the same communities, households, and individuals). In the context of nutrition-sensitive agriculture (NSA), co-location could imply enlisting a cadre of nutrition workers to provide nutrition counselling in the same communities and to the same households reached by agricultural extension agents. Integration would typically involve more extensive coordination and management between the sectors. However, a light-touch option could be to embed nutrition ideas and engagement within the usual delivery of agricultural services – specifically, to train agriculture extension workers on delivering basic messages surrounding nutrition and good diets alongside their usual services.

There is limited evidence about the effectiveness of agricultural extension workers relative to designated nutrition workers in delivering nutrition-related content. An obvious concern is whether agricultural extension workers may have difficulty learning, communicating, and tailoring this unfamiliar material. An additional consideration relates to gender. Agricultural extension workers tend to be male in many settings, while nutrition messaging is often targeted to women. If gender-based homophily matters for learning or adoption of nutrition practices, male extension workers could be less effective in communicating nutrition messages to women. To the best of our knowledge, existing research does not test whether the gender of the person delivering nutrition messages matters for uptake of content on nutrition by men and women. However, a substantial literature documents the role of gender in uptake of extension services (see Appendix A).^1^ Indeed, hiring more women agricultural extension workers has often been justified based on concerns around communications bottlenecks to female farmers, due to traditional and religious practices (such as *purdah*, or female seclusion) or women's lack of self-confidence in talking about their circumstances and problems with men (Lahai et al., 1999). Gender norms may also shape the type and content of extension messages that are trusted and perceived as appropriate (Feldstein et al., 1989, cited in Lahai et al., 1999). For instance, male knowledge providers may be less comfortable or credible to women regarding topics like breastfeeding, maternal nutrition, or food preparation, particularly in the South Asian context.

There are also potential benefits to having agricultural extension agents deliver nutrition content. In addition to lower cost and less coordination required to ensure content on agriculture and on nutrition reach the same individuals, gender-based homophily could contribute to male farmers being more likely to trust or value male agricultural extension agents’ information on nutrition. In settings where men play important roles deciding on food production and purchases, there may be benefits to engaging men to improve nutrition. Other differences between traditional nutrition workers and agricultural extension workers could also contribute to differing effectiveness in delivering nutrition information. For example, in many settings, agricultural extension agents tend to be more educated and better-compensated than nutrition workers.

In this study, we use a randomized controlled trial (RCT) to compare two models of delivering nutrition messages: deploying nutrition workers versus training agriculture extension workers to deliver basic nutrition messages. Our analysis is based on the Agriculture, Nutrition, and Gender Linkages (ANGeL) project in rural Bangladesh, implemented by the Ministry of Agriculture of the Government of Bangladesh. ANGeL was designed by the International Food Policy Research Institute (IFPRI) to inform scalable approaches for gender- and nutrition-sensitive agriculture in rural Bangladesh and compared different packages of interventions provided jointly to husbands and wives. Intervention components included agricultural training, nutrition behavior change communication (BCC), and gender sensitization trainings.

In this paper, we focus on comparing two treatment arms within ANGeL that were devoted to nutrition BCC, where the same nutrition content was provided either by (mostly male) sub-assistant agricultural officers (SAAOs) – also referred to as agricultural extension agents – who are permanent employees of the Department of Agricultural Extension (DAE) under the Ministry of Agriculture or by female nutrition workers. The female nutrition workers were hired from localities where ANGeL was implemented, specifically for the ANGeL project, and called “ANGeL *Pushti Kormi*” (APK; *Pushti Kormi* means “nutrition worker”) to distinguish them from other community nutrition workers such as those employed at the larger *upazila*- (subdistrict) or district-level by BRAC or the Ministry of Health and Family Welfare. In both treatment arms, the trainer (either the SAAO or the APK) provided 19 nutrition training sessions over a 17-month period. Each training site invited about 25 pairs of husbands and wives in participant farm households. Training sessions were interactive, including lectures as well as discussions, practical demonstrations, and question–answer sessions. Although agriculture topics were not formally part of the curriculum in these treatment arms, due to the interactive nature of the training, any topic raised by participants was discussed, including practicalities of how to produce the nutritious foods being promoted (Ahmed et al., 2022). Similarly, gender sensitization was not part of the curriculum in these arms; however, it is plausible that changes in empowerment and attitudes could have occurred due to men and women being brought together in groups on domains traditionally associated with the opposite gender (Brody et al., 2015, Quisumbing et al., 2021).

We thus compare effectiveness of nutrition training delivered by SAAOs versus APKs across several categories of outcomes for program participants: men’s and women’s knowledge of nutrition and agriculture, as well as adoption of improved agricultural practices; households’ production diversification, including whether they grew nutrient-rich foods highlighted in the training; households’ consumption of nutrient-rich foods highlighted in the training; households’ measures of diet; individual men’s and women’s measures of diet; and men’s and women’s empowerment and attitudes. We find no statistically significant difference between the outcomes depending on whether APKs or SAAOs provided the training, except for attitudes, where same-sex agents showed different effects on scores from opposite-sex agents. We further examine whether a co-resident mother-in-law (MIL) affects the effectiveness of each type of extension worker. Our results suggest that MIL presence attenuates the impact of the APK relative to the SAAO treatment on outcomes related to diet, women’s empowerment, and attitudes.

Our analysis provides insight into how outcomes related to nutrition, agriculture, and gender would change if agricultural extension workers provided nutrition information rather than traditional nutrition workers. The absence of a significant difference suggests that embedding nutrition content within agricultural extension workers’ services could be a plausible alternative to co-locating specialized nutrition workers with agricultural extension workers. However, an important caveat is that we do not study a model where both nutrition *and* agricultural information are explicitly part of the curriculum taught by both agricultural extension and traditional nutrition workers. Our findings regarding the differential impacts, conditional on the presence of the MIL also suggest that, in contexts where extended families are prevalent and in-laws may be influential decisionmakers in the household, extension delivery may need to consider intrahousehold and intergenerational dynamics.

Our study relates most closely to Olney et al. (2015), who also compare effectiveness of different types of providers for delivering nutrition information. They randomize the provision of nutrition BCC through either health committees composed of both men and women or older women leaders in a homestead food production program in Burkina Faso. They find that health committee members are better able to improve outcomes related to children’s nutritional status and dietary diversity compared to the older women leaders. They attribute the differences in impacts to differences in knowledge, efficacy, or influence of the actors who delivered the BCC messages, and not their gender. Ragasa et al. (2019) examine the provision of both agricultural extension and nutrition messages in Malawi. They do not address the question of the gender of the extension worker but that of the recipient. They find that in households where a primary male and female adult are present, dietary diversity is higher if both the man and women received nutrition advice and if they both received market access advice, compared to if either of them received it alone. None of these studies address the policy-relevant question of whether (mostly male) agriculture extension agents can deliver nutrition BCC with the same effectiveness as women nutrition workers.

## 2 Interventions, study design, and data collection^2^

### 2.1 Study design and intervention details

ANGeL aimed to deliver interventions that can leverage agricultural growth to increase farm household incomes, improve nutrition, and enhance women’s empowerment in Bangladesh. A key feature of ANGeL was its use of SAAOs to deliver training in all but one of the treatment arms. Conventionally, in Bangladesh and elsewhere, nutrition training is provided by staff at health posts or by community nutrition workers employed either by governments or by non-governmental organizations. Like other countries in South Asia, agricultural extension agents are mostly male, whereas frontline nutrition workers are typically female, based on traditional perceptions that agriculture is a male domain, and nutrition female. Such staffing patterns also assume that female nutrition workers are better able to interact with mothers in delivering nutrition BCC.

To assess whether male extension agents deliver nutrition training as effectively as female nutrition workers, ANGeL included both a treatment arm with nutrition training delivered by SAAOs (T(SAAO)) and a treatment arm with nutrition training delivered by trained female nutrition workers who lived locally and were recruited specifically for the program (T(APK)).^3^ For the T(SAAO) arm, ANGeL drew on SAAOs already working in the relevant blocks. For the T(APK) arm, *upazila*-level DAE officials solicited applications from women who completed at least secondary schooling in the 25 ANGeL blocks, interviewed the candidates, and hired the top individuals as APKs. The criteria and process for recruiting APKs followed usual local practices for recruiting community nutrition workers. Compensation for the two roles also followed typical patterns: APKs were paid 3,000 taka per month (consolidated), whereas SAAOs’ salaries ranged from 25,000–38,630 taka per month (based on salary scale) plus other allowances and pension after retirement. SAAOs were also paid 500 taka remuneration per ANGeL training session, while APKs did not get any remuneration for training besides salary.

For both the T(SAAO) and T(APK) arms, Helen Keller International (HKI) developed the curriculum and training materials for the nutrition BCC with the Bangladesh Institute of Research and Training on Applied Nutrition (BIRTAN) and IFPRI. Instructors from HKI trained APKs and SAAOs together on nutrition BCC at a Ministry of Agriculture facility near Dhaka; the form, content and duration of training was the same for both groups except on refresher training. Both SAAOs and APKs received three days intensive training on nutrition BCC, and both received printed training manuals: SAAOs received one day of refresher training; APKs received three days of refresher training.

Couples recruited for the study were invited to 19 nutrition BCC sessions over a 17-month period, delivered by either SAAOs or APKs depending on the treatment arm. The BCC sessions were conducted from July 2016 to December 2017, and each training session lasted approximately 1.5 h. Training took place either in meeting rooms or open courtyards in the villages where study participants resided; approximately 90 percent of participants reported that training sites were within one kilometer of their homes. Trainings included lectures, interactive discussions, practical demonstrations, and question–answer sessions. Both husbands and wives were expected to attend each session, and active participation from both men and women was encouraged. Participants received a small allowance for each training session to cover incidental costs of attending: 125 taka for one participant or 250 taka per household if both the husband and wife participated. Appendix Table S1 summarizes the topics covered and the model of delivery.

### 2.2 Randomization, sampling, and survey administration

ANGeL’s sample was designed so that, relative to the control group, there was a sufficient sample size to detect impacts of a 10 % increase in households’ per capita daily calorie availability and the Women’s Empowerment in Agriculture Index (WEAI) score (Alkire et al., 2013), setting 80 % power and 0.05 level of significance. Power calculations used data from the 2011/2012 round of the Bangladesh Integrated Household Survey, which is nationally representative of rural Bangladesh. This sample size also provided 80 % power at 0.05 level of significance to detect an increase of one new food produced in homestead gardens and 7.5 % increase in a household-level Global Diet Quality Score – measures we use to assess impacts on production diversity and diets.

Because training would be conducted by SAAOs and APKs each assigned to a “block,” cluster-randomization was conducted at the block level. Working with the Ministry of Agriculture, we identified all rural *upazilas* that were agro-ecologically suitable for agricultural diversification and had good market connectivity, thus considered appropriate for the ANGeL interventions. From a list of 484 such *upazilas*, 16 *upazilas* were purposively selected, such that each of the eight administrative divisions of Bangladesh was represented. From the list of all 525 blocks in 16 *upazilas*, we randomly selected 10 blocks from each *upazila*, yielding 160 blocks. Based on the power calculations, these were randomly assigned as follows: 25 blocks to each treatment arm – T(SAAO), T(APK), as well as the additional treatments described in footnote 3 – and 35 blocks to the control group. One village from each block was randomly selected. Within each of these villages, 25 farm households with at least one child under 24 months were randomly selected to participate. This yielded 625 households in each treatment arm (1,250 households in total) and 875 households in the control group, for a total sample of 2,125 households.

Baseline data were collected between November 2015 and January 2016, prior to the start of the nutrition BCC sessions. Endline data were collected between January and March 2018, after BCC sessions had ended– ensuring minimal seasonal difference between baseline and endline surveys. In each household, both the primary female beneficiary and primary male beneficiary were interviewed separately. Some modules were answered by only the male (e.g., household demographics, assets and wealth, agricultural production), some were answered by only the female (e.g., food consumption and food security, diet data, women’s status, and decision-making autonomy) and some were answered separately by each (e.g., data needed to construct measures of knowledge, empowerment).

### 2.3 Outcome variables

We assess impacts on a set of outcome variables based on ANGeL’s theory of change; see Ahmed et al. (2022). ANGeL’s nutrition training focused on the importance of diverse diets, including micronutrient-rich foods and animal source foods. If the training improved knowledge, this could change consumption of these foods in three, non-mutually exclusive ways. Households might choose to: (1) begin or increase production of specific micronutrient-rich or animal-source foods: non-rice crops (from the field or homestead), milk, eggs, fish; (2) consume a greater quantity of these nutrient rich foods already being produced; and/or (3) re-allocate spending to increase consumption of these foods. This change could entail consumption of food groups that the household otherwise might not have consumed, or more frequent consumption of certain food groups. It could also increase the actual quantities consumed of various food groups, rebalancing toward those that are micronutrient-rich (e.g., fruits and vegetables) and derived from an animal source (e.g., eggs, milk, fish). Lastly, as the nutrition training emphasizes the importance of improving dietary quality for young children and for women of reproductive age, we assess whether individual diets were affected by these treatment arms, as well as whether the engagement with couples increased men’s and women’s empowerment within the household. Our outcome variables trace out this trajectory.

Appendix Table S2 summarizes our outcome variables. To assess whether ANGeL increased male and female participants’ nutrition knowledge, we administered questions related to the BCC curriculum – on optimal child feeding practices, the identification of foods rich in micronutrients, and correct food preparation practices. We also assessed participants’ knowledge of production practices related to micronutrient-rich foods – specifically, improved crop production practices, improved livestock and poultry rearing practices, and improved cultured fishpond practices. Scores on both tests were converted to percent scores. We also asked whether men and women had adopted a series of improved agricultural practices.

We assess whether the ANGeL treatment arms affected which crops participant households grew in fields and on homestead plots near homes. These measures include the Simpson Diversification Index (SDI), which has been used to assess production diversity in Bangladesh (Gautam and Faruqee, 2016, Rahman, 2009) and the number of non-rice field crops (Sibhatu and Qaim, 2018). We examine the impact of ANGeL on the number of non-rice field crops (grown on agricultural fields), and homestead crops.^4^ We distinguish between field crops and production on homestead gardens, as the latter (homestead vegetable and fruit production to meet micronutrient needs) was encouraged during the nutrition trainings. We consider assessed levels of production of fruits and vegetables on homestead gardens, whether the household produced any of the animal source foods emphasized in training - eggs, milk, fish – and the amount of animal source foods produced.

We assess impacts of ANGeL on food consumption in several ways, reflecting the different pathways from production to consumption. The first measure examines the most direct pathway, consumption out of own production. We assess annual homestead vegetable consumption, homestead fruit consumption, and the quantities of egg, dairy, and fish consumed out of own production in kilograms. Next, we consider household-level measures of consumption quantity. Using data from a seven-day recall of household food consumption, we calculate a Household Diet Diversity Score (HDDS) and per capita caloric availability. We also adapt a recently developed indicator, the Global Diet Quality Score (GDQS; Bromage et al., 2021), to assess household-level diet quality. GDQS is defined at the individual-level, wherein each respondent receives points for each GDQS food group, according to the quantity of consumption consumed for that food group during the 24-hour reference period. We adapt the GDQS to a household-level GDQS (hGDQS), analyzing household consumption of the food groups found in the GDQS over the 7-day recall period, then converting these to a daily adult equivalent.^5^

The ANGeL study also collected twenty-four-hour individual dietary recall data. In each household, the female in charge of food preparation (usually, the spouse of the household head) was interviewed about the foods consumed within and outside the home the previous day by all household members. Data on ingredients used to prepare meals, the caloric content of the foods prepared using food composition table specific to Bangladesh (Shaheen, 2013), and the portion size (grams) consumed by each household member were used to calculate caloric intakes for individuals aged 15 years and older. We also calculated Caloric Adequacy Ratios that assess caloric intakes relative to requirements and individual-level GDQS.

ANGeL also aimed to empower women, motivated by the documented links between empowerment status, agricultural production diversity, and nutritional outcomes in Bangladesh (Sraboni et al., 2014). Our measure of women’s empowerment at endline is the pro-WEAI, an additive and decomposable index based on the Alkire-Foster methodology adapted from the WEAI (Alkire et al., 2013) for use in agricultural development projects (Malapit et al., 2019). We use the individual empowerment score and the individual’s empowerment status. We are also interested in whether either treatment improved household gender parity and affected women’s and men’s attitudes about their own roles and gender norms, for which we constructed an attitudes score.

## 3 Methods and empirical methodology

### 3.1 Estimation strategy

We estimate intent-to-treat (ITT) impacts using an ANCOVA specification (McKenzie, 2012):

(1) $$Y_{\mathrm{ibt}}=\alpha_{t}+{\beta_{Y}Y}_{\mathrm{ibt}-1}+\beta_{T1}{T(SAAO)}_{b}+\beta_{T2}T({\mathrm{APK})}_{b}+\beta_{X}X_{\mathrm{ibt}-1}+\varepsilon_{\mathrm{ibt}}$$

where $Y_{\mathrm{ibt}}$ is the outcome of interest for individual *i* residing in block *b* at time *t*; $Y_{\mathrm{ibt}-1}$ is the outcome in the prior period (baseline); ${T(SAAO)}_{b}$ and ${T(APK)}_{b}$ are dummy variables that take the value of 1 if block *b* was assigned to nutrition education and training through SAAOs and APKs respectively, and takes the value of 0 otherwise; $X_{\mathrm{ibt}-1}$ is a vector of baseline covariates; and $\varepsilon_{\mathrm{ibt}}$ is an error term. $\beta_{T1}$ and $\beta_{T2}$ represent the single-difference impact estimator for SAAO and APK respectively. For outcomes of interest collected only at endline (such as knowledge of correct agricultural practices), we use single difference estimates that do not include baseline values of the outcome variables.

We include the following baseline covariates, intended to capture demographic and socioeconomic characteristics, human capital, land and labor availability, as well as access to information prior to intervention: age of household head, sex of household head, mean education level of household males age 18 and older, mean education level of household females age 18 and older, number of adults in the household, dependency ratio, household wealth index, whether the household had access to electricity, amount of land owned by the household, whether any fishponds were owned by the household, the number of mobile phones owned by the household, whether the household owned a television, whether the household had recently received an extension visit for crop production, whether the household had recently received an extension visit for livestock or fish production, and dummies for location (*upazila*). We also include a dummy variable if the household reported being adversely affected by the widespread flooding that occurred in Bangladesh in the 12-month period before the endline survey.

We estimate ordinary-least-squares regressions for outcome variables that are continuous and linear probability models for dichotomous outcomes. Our outcome variables relating to levels of specific types of foods produced and consumed (homestead vegetables, homestead fruits, eggs, dairy, fish) contain both many zero values and many very large values. For these outcomes, we use the inverse hyperbolic sine (IHS) transformation and report marginal effects following Bellemare and Wichman (2020). Our household and individual measures of diet (except for the HDDS) are log transformed. In all cases, standard errors are clustered at the block level, the unit of randomization. We conduct Wald tests to assess whether the difference in impacts estimated from T(SAAO) and T(APK) are statistically significant.

We assess robustness in three ways. First, we estimate equation (1) excluding baseline control variables. Second, to assess whether our results are robust to considerations relating to multiple hypothesis testing, we calculate Romano-Wolf (Romano and Wolf, 2005) stepdown adjusted p-values using the Stata rwolf2 routine (Clarke et al., 2020). Third, we test whether our results are robust to various types of disaggregation (demographics (number of adults; women’s education, presence of MIL; land available (size of homestead garden; land operated by the household); wealth index; and whether the household lived in an *upazila* affected by severe flooding.

### 3.2 Estimation sample, attrition, and baseline descriptives

We begin with the 2,125 households that comprised the sample at baseline of households in the two treatment groups and the control group. At endline, we successfully re-interviewed 2,069 households, representing 2.6 percent of the target baseline sample lost to follow up. Appendix Table S3 reports how attrition is correlated with treatment arm and baseline covariates. Coefficients on the treatment arms are small in magnitude. There is no statistically significant impact on attrition of either treatment arm. An F test shows that we cannot reject the null hypothesis that, jointly, attrition does not differ across treatment arms; the p-value for this test is 0.23. With respect to our baseline covariates, attrition was slightly higher in wealthier households and in households that had received an extension visit related to crop production in the 12 month period prior to the baseline survey. It was lower in households that owned a television and in households residing in *upazilas* where flooding had occurred in the 12-month period prior to the survey. Attrition is not significantly associated with other selected baseline covariates.

Table 1 reports the mean values for the baseline covariates included in our regressions. Household heads in the control group are, on average, 41 years old and are overwhelmingly male (three percent of heads are female). Males aged 18 or older have on average, 4.7 years of schooling and females have 5.2 years of schooling. Just over a quarter of control households have a fishpond and they operate 1.07 acres of land. In the 12 months prior to the baseline survey, 19 percent of households had received a visit from an extension officer relating to crop cultivation and six percent had received a visit from an extension officer relating to livestock, poultry, or fish production. Magnitudes of baseline covariates are similar across treatment and control arms, although there are small differences; Appendix Table S4 shows formal tests of balance. We include baseline covariates in our regressions to help account for these small differences.

**Table 1:** Sample characteristics.

	T(SAAO)		T(APK)		Control		All	
	Mean	SD	Mean	SD	Mean	SD	Mean	SD
Baseline values								
Age, household head	40.15	14.00	38.85	12.92	41.17	13.87	40.19	13.66
Household head is female	0.04	0.19	0.03	0.18	0.03	0.18	0.03	0.18
Average grades school, men 18y or older	4.87	3.65	4.50	3.71	4.72	3.89	4.70	3.77
Average grades school, women 18y or older	5.16	2.73	5.09	2.86	5.16	2.90	5.14	2.84
Number of adults	3.27	1.61	3.00	1.40	3.17	1.43	3.15	1.48
Dependency ratio	0.96	0.63	0.98	0.61	1.00	0.62	0.98	0.62
Wealth index	−0.04	2.64	−0.50	2.58	0.23	2.51	−0.06	2.59
Household has fishpond	0.24	0.43	0.20	0.40	0.27	0.45	0.24	0.43
Land operated (ha)	1.18	1.29	1.08	1.28	1.07	1.08	1.10	1.21
Mobile phones owned, number	1.77	1.27	1.54	1.30	1.62	1.22	1.64	1.27
Household owns television	0.36	0.48	0.24	0.43	0.36	0.48	0.33	0.47
Received extension visit related to crops	0.21	0.41	0.19	0.39	0.19	0.40	0.20	0.40
Received extension visit related to livestock, poultry, fish	0.03	0.16	0.05	0.21	0.06	0.24	0.05	0.21
Household has electricity	0.70	0.46	0.60	0.49	0.76	0.43	0.69	0.46
Shocks between baseline and endline								
Experienced flooding	0.64	0.48	0.60	0.49	0.74	0.44	0.67	0.47

## 4 Results

### 4.1 Characteristics of SAAOs and APKs

Table 2 describes characteristics of SAAOs and APKs. Virtually all SAAOs were men (92 percent), and all APKs were women (100 percent). SAAOs were older than APKs on average: 43 years of age compared to 31 years for APKs. Most SAAOs (57.9 percent) had completed some form of secondary school compared to only 16 percent of APKs. As government staff, SAAOs were permanent employees of the Ministry of Agriculture, receiving much higher pay than the APKs who were temporary employees hired for the ANGeL project. Both SAAOs and APKs reported completing a similar number of training sessions with similar numbers of women and men. At endline, we administered a 24-item test to both SAAOs and APKs on the material that they were teaching; mean scores on this test were high for both groups with little difference between them (84.2 percent for SAAOs; 85.0 percent for APKs).

**Table 2:** Comparison of SAAO and APK characteristics.

	SAAO	APK
Sex, percent		
Male	92.0	0.0
Female	8.0	100.0
Mean age, years	43.3 (9.6)	30.6 (6.7)
Education, percent		
Lower secondary	15.8	32.0
Upper secondary	26.3	52.0
Bachelor’s degree or higher	57.9	16.0
Religion, percent		
Muslim	83.3	80.0
Hindu or Christian	16.7	20.0
Ethnicity, percent		
Bangla	95.8	92.0
Hindi	4.2	8.0
Employment	Permanent government employee, Department of Agricultural Extension (DAE), Ministry of Agriculture	Locally recruited for ANGeL
Salary	25,000–38,630 taka per month (based on salary scale) plus other allowances and pension after retirement	3,000 taka per month (consolidated)
Remuneration for training sessions	500 taka per session per group	No additional remuneration
Prior occupation, percent		
Teacher	20.8	0.0
Other government job	4.2	0.0
NGO	8.3	16.0
Other occupation	16.7	32.0
Student	37.5	12.0
Not employed	12.5	40.0
Mean number of training sessions completed	20.4 (6.5)	17.7 (3.3)
Mean number of women that should attend training	23.6 (3.0)	23.4 (2.8)
Mean number of men that should attend training	23.4 (3.2)	23.3 (3.9)
Mean score on test of nutrition knowledge (percent)	84.2	85.0

Notes: Standard deviations in parentheses.

### 4.2 Implementation fidelity, design

Fidelity of implementation—whether the program was implemented as designed – was high in both treatment arms; see Appendix Tables S5–S8. Women attended 82 percent of the sessions provided by SAAOs and 86 percent of sessions run by APKs (the difference is significant at p < 0.01). Men attended 72 percent of SAAO sessions and 70 percent of APK sessions. In both treatment arms, more than 90 percent of men and women attended their training sessions together. However, if a husband refused to go, it was more likely that a woman could go by herself to a training session run by an APK (53.3 percent) than one led by a SAAO (46 percent).

Training sessions were held in a location approximately 0.5 km from participants’ homes, about a 10-minute walk. Participants reported valuing the training they received (Appendix Table S6). Nearly all respondents felt that the contents of the training sessions were moderately or very informative; around 80 percent described the trainers as very communicative, understandable, and well prepared (83 percent). More than 80 percent of participants reported that they mostly or always understood what was taught, and over 90 percent reported that if they did not understand what was taught, they asked the trainer to repeat, and the trainer did so happily. There are no meaningful differences in this assessment of trainings provided by SAAOs or APKs.

Both women (Appendix Table S7) and men (Appendix Table S8) reported that the training was helpful, whether provided by SAAOs or APKs. Trainings were perceived to be valuable in terms of both information learned and improved confidence, relationships, and social ties. Women in both arms reported that sessions improved their understanding of care and nutrition of women and children. Following the training, more than 70 percent of women in both arms reported that they gained more respect or status within their homes and communities and that they felt more confident in making decisions about spending money. Nearly all women reported forming close ties with other participants and meeting with new friends after the training. Similarly, men reported that trainings improved their understanding of care and nutrition of women and children and learned new agriculture practices. 70–75 percent of men reported gaining more respect or status within their homes and communities and feeling more confident in making decisions about spending money. More than 80 percent of men formed close ties with other participants, and more than 78 percent met with new friends after the training. Nevertheless, between 24 and 31 percent of women reported that participation in the program interfered with domestic responsibilities, as did 48–61 percent of men.

### 4.3 Impacts on knowledge

Table 3 reports the impact of the SAAO and APK treatment arms on knowledge of optimal nutrition practices and on improved agricultural practices relating to crops, livestock, and fish, and whether adoption of these improved practices differed by sex.

**Table 3:** Impacts on nutrition knowledge, agriculture knowledge and adoption of improved agricultural production practices, by sex.

	(1)	(2)	(3)	(4)	(5)	(6)	(7)	(8)
	Nutrition knowledge, percent correct	Agriculture knowledge, percent correct	Any adoption, improved agricultural practices	Number, improved agricultural practices	Nutrition knowledge, percent correct	Agriculture knowledge, percent correct	Any adoption, improved agricultural practices	Number, improved agricultural practices
	Women				Men			
Treatments								
T(SAAO)	3.257***	4.608***	0.094***	0.502***	4.894***	7.351***	0.244***	0.868***
	(0.663)	(1.201)	(0.031)	(0.159)	(0.698)	(1.078)	(0.037)	(0.157)
T(APK)	4.162***	3.434***	0.075***	0.274**	4.240***	6.312***	0.182***	0.711***
	(0.671)	(1.007)	(0.026)	(0.112)	(0.781)	(0.973)	(0.037)	(0.158)
P values on equality of treatments								
T(SAAO) = T(APK)	0.21	0.33	0.56	0.19	0.38	0.29	0.13	0.34
Mean, Control group	80.1	51.2	0.27	0.85	71.5	53.7	0.20	0.71
Observations	2,060	2,069	2,061	2,061	1,638	1,929	1,929	1,929
R-squared	0.167	0.266	0.223	0.233	0.198	0.255	0.199	0.189

Notes: Estimates are intent-to-treat from OLS models. Standard errors adjusted for clustering at block level are in parentheses. *p < 0.10; **p < 0.05; ***p < 0.01. All specifications include as independent variables the treatment indicators, baseline values for the outcome variable (except for those outcomes relating to agricultural knowledge and practice) and the following control variables: age and sex of household head, mean education levels of males and females 18 and older, number of adults, dependency ratio, wealth index, land owned at baseline, fishpond owned at baseline, baseline access to information as measured by (baseline) number of mobile phones owned, ownership of television, received extension visit for crop production, received extension visit for livestock or fish production, household has access to electricity, and baseline upazila.

Both treatment arms improved women’s nutrition knowledge. The magnitude of the impacts, however, was relatively small, possibly because knowledge was already relatively high, with women in the control group scoring 80 percent on the baseline test. The magnitude of the impacts on men’s knowledge was slightly higher, possibly because their baseline levels of knowledge were lower. There is no statistically significant difference in impact by treatment arm, nor are the impacts appreciably different between women and men.

Although the nutrition BCC curriculum did not explicitly emphasize training on agricultural topics, as noted above, any topic raised by participants was discussed due to its interactive nature, including practicalities of how to produce the nutritious foods being promoted. Both women and men indicated that the trainings led to increases in post-intervention agricultural incomes and that they learned new agricultural practices (see Table 3 and Appendix Tables S7 and S8), with men in the SAAO treatment arm most likely to say this. Consistent with these statements, both treatment arms increased knowledge of improved agricultural practices. This was slightly more pronounced for men and for participants in the SAAO treatment arm. That said, the magnitudes of these differences are small. Table 3 also shows that both treatments increased both the likelihood and number of improved agricultural practices adopted by both women and men, with the effect sizes larger for men. (These are disaggregated into crops, livestock, and fish in Appendix Table S9.) However, there are no statistically significant differences in these impacts by treatment arm for either women or men.

### 4.4 Impacts on production diversity and levels

Neither treatment increased diversification of household food production as measured by the SDI, the number of non-rice field crops, the number of crops produced on homestead gardens, or the likelihood of fish production (Table 4). There are increases at the extensive margin for egg and dairy production; for the APK treatment arm, these effect sizes are 6.8 and 6.2 percentage points respectively. While statistically significant, we cannot reject the null that these impacts are equal to those found for the SAAO treatment.

**Table 4:** Impacts on diversification of agricultural products grown in fields and on homestead plots.

	(1)	(2)	(3)	(4)	(5)	(6)
	Diversification of crops grown in fields		Diversification of products produced at the homestead			
	Simpson Diversification Index	Number, non-rice field crops	Number, homestead garden crops	Any egg production	Any dairy production	Any fish production
Treatments						
T(SAAO)	0.006	0.035	0.005	0.034	0.023	0.004
	(0.019)	(0.082)	(0.125)	(0.026)	(0.020)	(0.030)
T(APK)	0.007	0.041	0.151	0.068***	0.062**	−0.001
	(0.020)	(0.091)	(0.103)	(0.026)	(0.024)	(0.024)
P values on equality of treatments						
T(SAAO) = T(APK)	0.97	0.96	0.39	0.22	0.11	0.89
Mean, Control group	0.20	0.68	1.8	0.76	0.32	0.58
Observations	1,825	2,069	2,069	2,069	2,069	2,069
R-squared	0.397	0.272	0.294	0.118	0.197	0.207

Notes: See Table 3.

We also considered the intensive margin of production diversification. Table 5 shows that the APK treatment arm increased the production of eggs and dairy products produced on homesteads. Expressed as a percentage, the impacts are large (51 and 38.0 percent, respectively); but given low baseline values, the magnitudes are relatively small. For example, for eggs, the percentage change is equivalent to (relative to the control group), an increase in annual household egg production of 35 eggs. We do not reject the null that the impacts on egg and dairy production are equal across treatment arms. These modest effects could be because households were selling, not consuming, these products, but there is no statistically significant impact of either treatment arm on gross sales revenues from eggs, dairy, or fish products (result available on request).

**Table 5:** Impacts on production and consumption of foods produced on homestead plots.

	(1)	(2)	(3)	(4)	(5)	(6)	(7)	(8)
	Production				Consumption			
	Fruit and vegetables	Eggs	Dairy	Fish	Fruit and vegetables	Eggs	Dairy	Fish
Treatments								
T(SAAO)	−0.058	0.169	0.139	0.080	−0.033	0.187	0.146	0.050
	(0.102)	(0.139)	(0.109)	(0.125)	(0.091)	(0.123)	(0.100)	(0.118)
T(APK)	0.110	0.409***	0.320**	0.136	0.106	0.385***	0.311**	0.105
	(0.100)	(0.132)	(0.130)	(0.105)	(0.097)	(0.114)	(0.123)	(0.095)
		[0.51]	[0.38]			[0.47]	[0.36]	
P values on equality of treatments								
T(SAAO) = T(APK)	0.20	0.13	0.17	0.67	0.25	0.13	0.19	0.65
Mean, Control group (Levels)	209.9	69.7	78.5	179.8	125.2	44.9	37.8	94.9
Observations	2,069	2,069	2,069	2,069	2,069	2,069	2,069	2,069
R-squared	0.277	0.147	0.211	0.311	0.273	0.148	0.203	0.274

Notes: See Table 3. Values in square brackets are marginal effects.

### 4.5 Impacts on food consumption

Given that both treatment arms led to increased quantities of certain foods produced on the homestead, we assess the extent to which study participants consumed this increased production. Columns 5–8 of Table 5 indicate that the APK treatment resulted in a statistically significant increase in consumption of eggs and dairy, but not fruits, vegetables, or fish. The magnitudes of these effect sizes are large when expressed as percent increases – 47 percent for eggs and 36 percent for dairy – but again given the low baseline mean levels of consumption of these foods, the absolute level of the change is modest. The coefficients of the SAAO treatment arm on the consumption of eggs and dairy are positive, but not statistically significant and we cannot reject the null that they are equal to the coefficients for the APK treatment arm.

We now turn to three household-level measures of diet, the HDDS, caloric availability, and the hGDQS (Table 6). Both treatment arms increase household diet diversity, but the magnitudes are small relative to the baseline control group mean of 7.7 food groups: 0.16 for T(SAAO) and 0.33 for T(APK) Impacts on household calories are small and imprecisely measured. By contrast, when we use log hGDQS, both treatments have a significant effect, increasing this measure of dietary quality by 6.3 (SAAO) and 5.1 percent (APK); the difference in these impacts is not statistically significant.

**Table 6:** Impacts on measures of household diet.

	(1)	(2)	(3)
	Dietary Diversity Score	Log per capita caloric acquisition	Log household Global Diet Quality Score
Treatments			
T(SAAO)	0.163**	0.028*	0.061***
	(0.080)	(0.016)	(0.010)
		[0.028]	[0.063]
T(APK)	0.332***	0.020	0.050***
	(0.095)	(0.015)	(0.012)
			[0.051]
P values on equality of treatments			
T(SAAO) = T(APK)	0.08	0.66	0.43
Mean, Control group (Levels)	7.7	1982	22.2
Observations	2,069	2,069	2,069
R-squared	0.271	0.109	0.285

Notes: See Table 3. Values in square brackets are marginal effects.

In Table 7, we assess whether these changes in household diet benefit both men and women. There is no impact on caloric intake for either men or women, even after adjusting for caloric requirements. However, both treatment arms improve both women’s and men’s diet quality, by 5.5 percent for the T(SAAO) arm and 8.8 to 9.0 percent for the T(APK) arm with the effects nearly identical for women and men.

**Table 7:** Individual dietary intakes (calories, GDQS).

	(1)	(2)	(3)	(4)	(5)	(6)	(7)	(8)	(9)
	All			Males			Females		
	Log caloric intake	Log calorie adequacy ratio	Log Global Diet Quality Score	Log caloric intake	Log calorie adequacy ratio	Log Global Diet Quality Score	Log caloric intake	Log calorie adequacy ratio	Log Global Diet Quality Score
Treatments									
T(SAAO)	0.003	−0.003	0.054**	0.008	0.009	0.055**	−0.003	−0.011	0.055**
	(0.015)	(0.006)	(0.021)	(0.021)	(0.007)	(0.021)	(0.014)	(0.008)	(0.023)
			[0.055]			[0.056]			[0.056]
T(APK)	0.002	−0.001	0.089***	−0.004	0.004	0.090***	0.005	−0.004	0.088***
	(0.013)	(0.006)	(0.020)	(0.016)	(0.005)	(0.022)	(0.013)	(0.009)	(0.021)
			[0.093]			[0.094]			[0.091]
P values on equality of treatments									
T(SAAO) = T(APK)	0.96	0.74	0.18	0.60	0.42	0.23	0.58	0.48	0.21
Mean, Control group (Levels)	2354	0.90	8.18	2488	0.86	8.36	2232	0 0.94	8.01
Observations	5,490	5,490	5,490	2,501	2,501	2,501	2,989	2,989	2,989
R-squared	0.19	0.77	0.21	0.16	0.78	0.21	0.15	0.75	0.21

Notes: See Table 3. Values in square brackets are marginal effects.

### 4.6 Impacts on empowerment and attitudes

Table 8 presents single-difference ITT impacts of the SAAO and APK treatments on pro-WEAI outcomes: women’s and men’s empowerment scores, whether women and men are empowered, and whether the household achieves gender parity. In the control group at endline, the mean empowerment score for women is 0.59; only 25 percent of women are empowered, compared to 39 percent of men, and 47 percent of control households achieve gender parity. For women’s empowerment outcomes, there are significant positive impacts from both treatment arms relative to the control group. The women’s empowerment score increases by 0.03 and the prevalence of empowered women increases by 5–6 percentage points. For both outcomes, Wald tests show that there is no statistically significant difference in impacts by treatment arm. The impacts on men’s empowerment are comparable in magnitude, with no statistically significant differences by treatment arm.

**Table 8:** Single-difference impacts on Pro-WEAI and on attitudes score.

	(1)	(2)	(3)	(4)	(5)	(6)	(7)
	Women			Men			
	Empowerment score	Whether empowered	Total attitudes score	Empowerment score	Whether empowered	Total attitudes score	Gender parity
Treatments							
T(SAAO)	0.035***	0.053**	0.433	0.031***	0.089***	0.609**	0.020
	(0.011)	(0.026)	(0.265)	(0.010)	(0.032)	(0.248)	(0.029)
T(APK)	0.034***	0.066**	0.721**	0.027***	0.084***	−0.010	0.018
	(0.011)	(0.029)	(0.298)	(0.009)	(0.031)	(0.195)	(0.032)
P values on equality of treatments							
T(SAAO) = T(APK)	0.94	0.67	0.34	0.67	0.88	0.03	0.97
Mean, Control group (Levels)	0.59	0.25	34.4	0.67	0.39	34.5	0.47
Observations	1,743	1,743	1,743	1,743	1,743	1,743	1,743
R-squared	0.123	0.068	0.151	0.131	0.089	0.082	0.082

Notes: See Table 3. Estimates are single difference. Sample is restricted to households where both women and men complete the survey modules needed to construct the Pro-WEAI.

When we focus on the attitudes score, a slightly different pattern emerges. The SAAO treatment increases the attitudes score more for men whereas the APK treatment increases this more for women; the difference in impacts between the SAAO and APK treatments for men is statistically significant. That said, the magnitude of the impact of the SAAO treatment on men’s attitudes is small, 0.60, relative to the control group mean of 34.5, and appears to be driven by responses to a few items that make up the score. Appendix Table S10 presents impacts on the individual items that comprise the attitudes score, separately for women and men. The lone item for which impacts were significantly different between SAAO and APK arms (p < 0.05) for women was related to not voicing one’s opinion for fear of being ignored or ridiculed. The estimated coefficient was negative and significant for women in the APK treatment arm (this item was reverse coded when computing the aggregate attitudes score). The two items for which the SAAO and APK arms have statistically different (at p < 0.05) impacts on men’s responses relate to men’s perception that they make positive contributions to their community (with a positive estimated coefficient) and that women are usually busy with work that benefits the household (although individual estimated impacts are not statistically different from zero).

### 4.7 Robustness checks

We subjected all results to three robustness checks. In Appendix Table S11, we show results when we exclude all control variables and baseline values, leaving only the dummy variables for treatment status as controls. We obtain parameter estimates nearly identical to those shown in Table 3, Table 4, Table 5, Table 6, Table 7, Table 8 but, predictably, these are estimated with less precision. In Appendix Table S12, we assess whether our results are robust to adjusting for multiple hypothesis testing across the outcome domains we consider in the paper; again, our results are robust to this concern. Testing whether our results are robust to various types of disaggregation –demographics (number of adults; women’s education, presence of MIL); land available (size of homestead garden; land operated by the household); wealth index; and whether the household lived in an *upazila* affected by severe flooding—shows that estimated impacts are, in most cases, indistinguishable across treatment arms (results available upon request), with one notable exception: whether the mother-in-law is coresident.

There is a mother-in-law (MIL) present in approximately 23 percent of the households in our sample. The presence of a MIL has no effect on knowledge acquisition or production of fruits, vegetables, or animal source foods (results available on request). However, there are differential effects of resident MIL on household consumption reducing the impact of the SAAO and especially the APKs (Table 9a). The treatment impact on log per capita caloric acquisition is significantly lower in households with resident MIL, regardless of treatment arm. If a MIL is present, the treatment impact on household GQDS is significantly lower in the APK treatment and there is no significant impact in the SAAO arm. Turning to impacts on individual diets, we do not detect significant effects of MIL presence when looking at a pooled sample of individuals (Table 9b), nor does MIL presence affect the impacts on male caloric intake, calorie adequacy, and GQDS (although impact estimates on GQDS of males are positive and significant in the SAAO arm when MIL is present, and positive and significant in the APK arm when MIL is not present). Although we observe positive and significant impacts on females’ GQDS in both treatments when the MIL is not present differences according to MIL presence are not statistically significant.

**Table 9:** (a) Impacts on measures of household diet, by presence of mother-in-law. (b) Impacts on measures of individual diets, by presence of mother-in-law. (c). Impacts on measures of empowerment and gender parity, by presence of mother-in-law.

(a)			
Treatments	(1)	(2)	(3)
	Dietary Diversity Score	Log per capita caloric acquisition	Log household Global Diet Quality Score
Mother-in-law NOT present			
T(SAAO)	0.119	0.049**	0.067***
	(0.087)	(0.019)	(0.012)
T(APK)	0.366***	0.039**	0.060***
	(0.107)	(0.016)	(0.012)
Mother-in-law present			
T(SAAO)	0.229	−0.026	0.043***
	(0.128)	(0.025)	(0.016)
T(APK)	0.236	−0.024	0.028
	(0.139)	(0.026)	(0.019)
P values			
T(SAAO), mother-in-law NOT present = T(SAAO), mother-in-law present	0.44	0.02	0.18
T(APK), mother-in-law NOT present = T(APK), mother-in-law present	0.40	0.02	0.05

(b)									
	(1)	(2)	(3)	(4)	(5)	(6)	(7)	(8)	(9)
	All			Males			Females		
	Log caloric intake	Log calorie adequacy ratio	Log Global Diet Quality Score	Log caloric intake	Log calorie adequacy ratio	Log Global Diet Quality Score	Log caloric intake	Log calorie adequacy ratio	Log Global Diet Quality Score
Treatments									
Mother-in-law NOT present									
T(SAAO)	−0.005	−0.008	0.045**	−0.010	−0.009	0.038	−0.001	−0.006	0.052**
	(0.016)	(0.016)	(0.022)	(0.021)	(0.021)	(0.024)	(0.014)	(0.016)	(0.024)
T(APK)	−0.004	−0.008	0.096***	−0.013	−0.024	0.097***	0.004	0.007	0.096***
	(0.014)	(0.015)	(0.020)	(0.017)	(0.019)	(0.023)	(0.013)	(0.015)	(0.021)
Mother-in-law present									
T(SAAO)	0.016	0.025	0.066**	0.048	0.047	0.097***	−0.012	0.006	0.044
	(0.026)	(0.028)	(0.033)	(0.035)	(0.038)	(0.036)	(0.027)	(0.030)	(0.035)
T(APK)	0.025	0.016	0.054	0.028	0.018	0.055	0.019	0.015	0.051
	(0.022)	(0.023)	(0.041)	(0.029)	(0.031)	(0.045)	(0.023)	(0.023)	(0.041)
P values									
T(SAAO), mother-in-law NOT present									
= T(SAAO), mother-in-law present	0.43	0.23	0.55	0.096	0.14	0.13	0.68	0.72	0.81
T(APK), mother-in-law NOT present									
= T(APK), mother-in-law present	0.25	0.35	0.30	0.22	0.23	0.36	0.56	0.78	0.29

(c)							
	(1)	(2)	(3)	(4)	(5)	(6)	(7)
	Women		Men				Gender parity
	Empowerment score	Whether empowered	Total attitudes score	Empowerment score	Whether empowered	Total attitudes score	
Mother-in-law NOT present							
Treatments							
T(SAAO)	0.05***	0.08**	0.33	0.04***	0.06	0.68**	0.04
	(0.01)	(0.03)	(0.26)	(0.01)	(0.04)	(0.30)	(0.03)
T(APK)	0.05***	0.09**	0.70**	0.04***	0.10***	−0.06	0.02
	(0.01)	(0.03)	(0.34)	(0.01)	(0.04)	(0.22)	(0.04)
Mother-in-law present							
Treatments							
T(SAAO)	−0.01	−0.02	0.40	0.01	0.15**	0.29	−0.05
	(0.02)	(0.04)	(0.56)	(0.02)	(0.06)	(0.37)	(0.05)
T(APK)	−0.01	0.01	0.47	0.00	0.05	0.15	0.01
	(0.02)	(0.04)	(0.52)	(0.01)	(0.06)	(0.43)	(0.06)
P values on equality of treatments							
T(SAAO), mother-in-law NOT present							
= T(SAAO), mother-in-law present	0.0050***	0.0640*	0.9010	0.2252	0.2526	0.3903	0.1265
T(APK), mother-in-law NOT present							
= T(APK), mother-in-law present	0.0099***	0.1097	0.6931	0.0391**	0.4480	0.6625	0.8194

Notes: See Table 3.

Notes: See Table 8.

Coresident MIL change the empowerment environment within the household. They significantly weaken program impacts of both SAAO and APKs on the women’s empowerment score, with a weak or undetectable impacts on whether women are empowered or the total attitudes score (Table 9c). While the MIL’s presence has no impact on the effectiveness of the SAAO treatment on men’s empowerment scores, it significantly weakens the impact of the APK treatment on men’s empowerment. No impacts of MIL presence are detected on the total attitudes score nor gender parity.

MILs’ presence appears to affect only some components of the attitudes score (Appendix Table S13) with some unexpected impacts. Women in both SAAO and APK treatments are less likely to agree with statements that women should stand up for themselves to get what they want if the MIL is present. Interestingly, women in SAAO treatments are likely to agree with statements that women are busy with work that helps the household and that husbands should help wives with household chores when the MIL is present. Men are more likely to agree that they make important contributions to the family and that they should help their wives in household chores in the SAAO treatment if their mothers are not present; they are more likely to agree that they make important contributions to the community in the SAAO treatment if their mothers are present.

## 5 Discussion and policy implications

Despite their different backgrounds and compensation, SAAOs and APKs seem to generate similar improvements in nutrition knowledge and good agricultural practices (even though agriculture training was not a part of the nutrition BCC), similar non-impacts on most measures of agriculture production diversity (eggs and dairy being the exceptions) and similar and relatively large improvements in hGDQS. Across many of these impacts, there is a slightly larger impact when the training is delivered by APKs but we generally cannot reject the null of equal effects. The only area where having the same gender as the trainer appears to have a greater impact is on attitudes: men’s attitude scores increase more when trained by male SAAOs, and women by female APKs. We note that while impacts on production expressed in percentage terms are often large, in absolute terms they are often more modest. While this suggests that nutrition BCC has the potential to improve production outcomes, it should not be seen as a panacea. It is, however, a potential model that can contribute towards better diets of rural households.

These effects, however, are modified when the MIL, typically the husband’s mother in Bangladesh, is present. While MIL presence does not appear to have differential impacts on production outcomes, MIL presence apparently weakens the effectiveness of nutrition trainings, with some differences across treatment arms. Impacts of the APK treatment on log per capita caloric acquisition and log household GQDS are less when the MIL is present; no difference is detected when considering individual diets. MIL presence weakens the positive impacts of nutrition BCC on women’s empowerment score in both the SAAO and APK treatments, and even attenuates the impact of the APK treatment on men’s empowerment scores. It is possible that the MIL counteracts the messaging given by a presumably younger female, the APK, on men’s empowerment, but regardless of the gender of the extension agent, the MIL dampens program impacts on women’s empowerment scores.

Although the attitudes score is not a component of the empowerment score, differential impacts on some items may help interpret the effect of coresident MILs. Notably, coresident MILs may not support changes in gender norms that challenge traditional patterns (men helping in the domestic sphere, contributing to the family) but go along with those that are consistent with cultural stereotypes (men’s contribution to the community). Interestingly, women with coresident MILs are more likely to say that women are busy with work that benefits the household and that husbands should help wives with household chores. This inconsistency may reflect mixed impacts of MIL presence, but also highlights their influence within the household.

Our findings indicate that in most cases, the effectiveness of mostly male agricultural extension workers in improving nutrition knowledge, agricultural knowledge, and women’s empowerment does not significantly differ from the effectiveness of the program’s female nutrition workers. This finding differs from conventional wisdom that same-sex agents are more effective in reaching women, as suggested by studies of agricultural extension in Africa (e.g., Kondylis et al., 2016, Buehren et al., 2019). However, our findings also suggest that these effects may be different in complex, intergenerationally extended households.

Several caveats apply to these findings. First, we do note a pattern of larger point estimates from APK training than SAAO training for increases in homestead production of eggs and dairy, household consumption of eggs and dairy, and men’s and women’s GDQS. However, differences between APK impacts and SAAO impacts on these outcomes are not statistically significant, thus not conclusive. Second, while we highlight the difference in gender composition of the SAAOs versus APKs, there are differences besides gender in these two groups that could play a role in their relative effectiveness. For example, SAAOs tend to hold higher education levels and were substantially better compensated; SAAOs participating in ANGeL were also experienced, while APKs were newly hired for this project. Thus, we do not compare two delivery modalities that differ only by gender. That said, the T(SAAO) and T(APK) arms are fairly representative of the types of staff who could be realistic options for delivering nutrition content in Bangladesh, thus the comparison is policy-relevant. Third, we note the differential impact of coresident mothers-in-law on estimated impacts. However, because the sample of households with coresident MILs is smaller relative to those without MILs present (438 vs. 1,299 in the empowerment analysis) we may be underpowered to detect impact. Nevertheless, our results suggest that gender alone does not fully explain intrahousehold dynamics; differences in status associated with the life-cycle and family position may also matter.

Bearing in mind these caveats, our results suggest opposite-sex agents may not necessarily be a barrier to effective training. Can training men and women jointly overcome the usual barriers faced in training those of a different gender? For example, because husbands were present, it is possible that male extension workers were more comfortable discussing nutrition topics in front of women. Although we cannot answer this question definitively, since we did not have a treatment arm where men or women were trained alone, this finding is consistent with several studies conducted in Africa. For example, Ragasa et al. (2019) find that, in Malawi, targeting agriculture and nutrition messages to husbands and wives together was more effective than targeting to individual spouses. Lambrecht et al. (2016) find that joint participation in an extension program on integrated soil fertility management in the Democratic Republic of Congo leads to the highest adoption rates compared to female or male participation alone. Similarly, in Uganda, Lecoutere et al. (2019) show that providing information to female and male co-heads together can contribute to greater involvement of women in joint decision-making and joint action even if they may not translate into better agricultural outcomes on jointly managed plots or increased joint sales.

Indeed, qualitative work on ANGeL reveals that men and women beneficiaries in the T(SAAO) and T(APK) arms valued the joint training of husbands and wives (Quisumbing et al., 2021). For example, a woman beneficiary in the APK treatment arm said (Younus 2018):

“If I attend the training sessions alone, I (have) to explain in detail to my husband. It can be tough for me to convince him. Now, since we go together, he knows all the things. We discuss and take decision easily.” — (Woman beneficiary, APK arm)

“It is very much helpful for the family if trainings are combined…Nutrition is from vegetables. Now, my wife grows vegetables at home to help meet our nutritional demands.” — (Man beneficiary, SAAO arm)

There are several features of the intervention that likely contributed to positive impacts. In both arms, the implementing frontline workers – the SAAOs and the APKs – are compensated for their work; the development of training materials and pedagogical approaches drew on expertise and experience around agriculture, nutrition, and gender; and both SAAOs and APKs were well-trained using the same training methods and trainers. On the demand side, the participating households received small incentives and the intervention deliberately targeted married couples. In addition, the delivery of the intervention content in groups, rather than via 1–1 interactions, is an important feature to consider – joint learning, sharing, support and peer pressure could all have contributed to the kinds of impacts found here. Thus, the ANGeL intervention itself, in all its fullness, was a well-designed and well-implemented intervention, and in this context, opposite-sex trainers did not prevent improvements in knowledge, practices, and nutrition-related outcomes.

The possibility of attenuated program impacts when a mother-in-law is coresident is consistent with other findings in Bangladesh and in South Asia more generally. White (2005) attributes the modest impact of the Bangladesh Integrated Nutrition Project party to a failure to recognize the importance of social pressures that constrain the adoption of improved practices. Programs that target nutrition information only to mothers assume that they are the most important decisionmakers on nutrition, but husbands and MILs are important decisionmakers as well. Other studies in South Asia have pointed to the influential role of MILs in household decisions, particularly those related to health and nutrition (Alam et al., 2020, Rasul et al., 2021), and even access to social networks (Anukriti et al., 2020). Even if ANGeL was designed to increase husbands’ support, which studies in Bangladesh have found important in maternal nutrition interventions (Nguyen et al., 2017), the potential influence of MILs in extended households suggests that including them in nutrition trainings might help to bring them on board, rather than have them offset the positive impact of nutrition BCC. Doing so, however, requires attention to ensuring that the content of the training resonates with MILs, as Wable Grandner et al. (2022) demonstrate.

## 6 Conclusion

Our study, based on a cluster-randomized controlled trial in rural Bangladesh, provides evidence on the effectiveness of alternative delivery workers in providing nutrition BCC to women and men, who were trained jointly. Both approaches increased nutrition knowledge of men and women, household and individual diet quality and women’s empowerment. We find no significant difference in men’s and women’s agricultural knowledge, nutrition knowledge, dietary diversity, women’s empowerment, and gender parity, whether the training was delivered by mostly male agriculture extension agents or female nutrition workers hired by the project. The only evidence of same-sex homophily comes from an attitudes score, which increases more for men if they were trained by SAAOs, and more for women if trained by APKs. However, the weakening of program impacts when MIL are present suggests that BCC strategies may need to be designed to explicitly target influential household members, especially if the extension agent may be perceived as a woman of lower status because of her age.

Our findings also appear to run counter to the conventional wisdom that farmers learn more from trainers of the same sex. However, those studies were conducted in Africa, where there is possibly a clearer delineation between men’s and women’s responsibilities in agriculture. Although it would be ideal to train more female extension workers, the realities of agricultural extension systems in South Asia are that the pipeline into government agricultural extension departments remain male-dominated. In the short run, to scale up nutrition-sensitive agriculture in South Asia, it is still important to train male agriculture extension workers to deliver nutrition-sensitive agriculture content effectively. ANGeL participants perceive that the provision of training to husbands and wives together was an important factor behind the effectiveness of this intervention; this is consistent with the growing popularity of “household methodologies” such as the Gender Action Learning System (GALS) where husbands and wives are trained together to visualize a future for their family and to plan towards that goal (IFAD, 2022). Modifying training modalities to account for other influential members within the household—such as mothers-in-law--is a potential area for future programming.

## Funding

We acknowledge funding from the 10.13039/100000200United States Agency for International Development (USAID) through the Policy Research and Strategy Support Program (PRSSP) in Bangladesh under USAID Grant Number EEM-G-00-04-00013-00; the Gender, Agriculture, and Assets Project – Phase 2 (GAAP2), supported by the 10.13039/100000865Bill & Melinda Gates Foundation through INV-008977; the CGIAR Research Programs on Agriculture for Nutrition and Health (A4NH) and Policies, Institutions, and Markets (PIM); and TAFSSA (the OneCGIAR research initiative on Transforming Agrifood Systems in South Asia).

## CRediT authorship contribution statement

**Akhter Ahmed:** Conceptualization, Investigation, Supervision, Project administration, Funding acquisition, Writing – original draft, Writing – review & editing. **Fiona Coleman:** Software, Formal analysis, Writing – original draft, Writing – review & editing, Methodology. **John Hoddinott:** Conceptualization, Investigation, Software, Formal analysis, Writing – original draft, Writing – review & editing, Methodology. **Purnima Menon:** Conceptualization, Investigation, Writing – original draft, Writing – review & editing. **Aklima Parvin:** Conceptualization, Investigation, Supervision, Project administration, Writing – original draft, Writing – review & editing. **Audrey Pereira:** Software, Formal analysis, Writing – original draft, Writing – review & editing, Methodology. **Agnes Quisumbing:** Conceptualization, Investigation, Writing – original draft, Writing – review & editing, Methodology, Funding acquisition. **Shalini Roy:** Conceptualization, Investigation, Writing – original draft, Writing – review & editing, Methodology, Funding acquisition.

## Declaration of Competing Interest

The authors declare that they have no known competing financial interests or personal relationships that could have appeared to influence the work reported in this paper.

## Footnotes

1 Less investigation of differences in the effectiveness of men or women in delivering nutrition messages seems to be based on the premise that nutrition is a woman’s domain. Most nutrition workers in many settings are women.

2 This section draws on Quisumbing et al (2021).

3 ANGeL included additional treatment arms that provided agricultural training on diversifying agriculture production, a treatment arm that combined agricultural training and nutrition BCC, and a treatment arm with agriculture, nutrition BCC, and gender sensitization. Analysis of the comparative effects of these treatments is found in Ahmed et al. (2022).

4 We exclude permanent tree crops such as mangoes and jackfruit, given the lag between planting these are their production of fruit.

5 Because of our adaptation, our household-level calculations of hGDQS are not directly comparable to the GDQS calculated at the individual-level based on 24-hour diet intake recall. However, because we construct hGDQS in a consistent manner across the intervention arms in this study, this should not introduce bias for assessing impacts.
