# Supplementary material for: Comparing delivery channels to promote nutrition-sensitive agriculture: A cluster-randomized controlled trial in Bangladesh
Source: Food Policy. 2023 Jul;118:102484. doi: 10.1016/j.foodpol.2023.102484 (PMC10398750; doi:10.1016/j.foodpol.2023.102484)
Supplement: Supplementary data 1 [file mmc1.docx]

**On-line Supplementary Appendix**

For

**Comparing delivery channels to promote nutrition-sensitive agriculture: A cluster-randomized controlled trial in Bangladesh**

31 May 2023

**Table S1: Description of ANGeL nutrition training**

|  | Nutrition training |
| --- | --- |
| Length of training period | 17 months |
| Number of sessions | 19 |
| Topics covered | - Functional roles played by different types of food - The importance of a balanced diet - Micronutrients (vitamin A, iron, iodine, and zinc) and sources of food containing these - Age-appropriate complementary foods - Optimal breastfeeding practice - Maternal nutrition and care - Safe food preparation and preservation, hygiene, and handwashing |
| Training format | Lectures, interactive discussions, games, and cooking demonstrations |
| Who was invited to attend | Husbands and wives |

**Table S2: Description of outcomes**

| Domain | Variable | Description | Notes |
| --- | --- | --- | --- |
| Knowledge | Nutrition knowledge, percent correct | Respondents (mothers and fathers of children aged < two years) were administered 20 questions on optimal feeding practices for children < 2 years, identification of foods rich in micronutrients such as Vitamin A, iron, and zinc, and optimal food preparation practices (for example, cooking vegetables with oil to improve absorption of fat-soluble vitamins). | Ranges in value from 0 to 100  At endline, less than five percent of respondents scored below five percent or greater than 95 percent. |
|  | Agriculture knowledge, percent correct | Respondents (women and male household members - usually husbands) were administered 32 questions covering cultivating fruit and vegetable crops, particularly in homestead gardens. Questions included: preparation of pits and beds for vegetable production, identifying quality seeds and fertilizers, seed storage, and organic methods of controlling pests. Similar questions were asked about the care and feeding of livestock and poultry and fish culture. | Ranges in value from 0 to 100  At endline, less than five percent of respondents scored below five percent or greater than 95 percent. |
| Adoption of new practices | Number, improved agricultural practices | Respondents (women and male household members - usually husbands) were administered 15 questions covering improvements to the management of their homestead gardens, livestock and poultry raising, and fishpond management. | Ranges in value from 0 to 15  At endline, less than two percent of respondents reported adopting 12 or more new practices |
|  | Any adoption, improved agricultural practices | Respondents (women and male household members - who were usually their husbands) were administered 15 questions covering improvements that could have made to the management of their homestead gardens, the raising of livestock and poultry or the management of fishponds. | =1 if any new practice was adopted, =0 otherwise |
| Production diversity on fields | Simpson Diversification Index (SDI) | The SDI accounts for both the number of different crops that the household grows, and the acreage devoted to different crops. A value of zero means that the household devotes all its land to one crop. Higher values (values closer to 1) imply greater crop diversity. | Ranges in value from 0 to 1 |
|  | Number, non-rice field crops | Number of non-rice crops grown in farmer fields |  |
| Production diversity on homestead | Number, homestead garden crops | Number of different fruit and vegetable crops grown in the homestead garden during the last 12 months |  |
|  | Any egg production | Whether poultry produced any eggs in the last 12 months | =1 if yes, =0 otherwise |
|  | Any dairy production | Whether dairy cows produced any milk in the last 12 months | =1 if yes, =0 otherwise |
|  | Any fish production | Whether household harvested any fish from fishponds in the last 12 months | =1 if yes, =0 otherwise |
| Production from homestead | Fruits and vegetables | Quantity (kg) of fruit and vegetables produced in homestead gardens in the last 12 months | Inverse Hyperbolic Sine (IHS) transformed |
|  | Eggs | Quantity (number) of eggs produced in the last 12 months | IHS transformed |
|  | Dairy | Quantity (litres) of milk produced in the last 12 months | IHS transformed |
|  | Fish | Quantity (kg) of fish produced in the last 12 months | IHS transformed |
| Consumption from homestead | Fruits and vegetables | Quantity (kg) of fruit and vegetables consumed out of production from homestead gardens in the last 12 months | IHS transformed |
|  | Eggs | Quantity (number) of eggs consumed out of own production in the last 12 months | IHS transformed |
|  | Dairy | Quantity (litres) of milk consumed out of own production in the last 12 months | IHS transformed |
|  | Fish | Quantity (kg) of fish consumed out of own production in the last 12 months | IHS transformed |
| Household diet | Household Diet Diversity Score (HDDS) | Uses data from seven-day recall of household food consumption. We determine whether (yes =1; no=0) households consumed foods from the following groups: Cereals; Roots and tubers; Vegetables; Fruit; Meat, poultry, offal; Eggs; Fish and seafood; Pulses, legumes and nuts; Milk and milk products; Oils/fats; Sugar/honey; Other foods. These values are summed to create the DDS. | Ranges in value from 0 to 12  At endline, less than five percent of respondents reported consuming more than 10 food groups |
|  | Per capita caloric acquisition | Using data from seven-day recall of household food consumption, convert calories available for consumption to a daily value and divide by household size. | Log transformed |
|  | household Global Diet Quality Score (hGDQS) | Uses data from seven-day recall of household food consumption. The GDQS consists of 25 food groups: 16 healthy food groups, 7 unhealthy food groups, and 2 food groups (red meat, high-fat dairy) that are unhealthy when consumed in excessive amounts. For 24 of the GDQS food groups, three ranges of quantity of consumption are defined (in grams/day): low, medium, and high. For one food group (high-fat dairy), four ranges of quantity of consumption are used: low, medium, high, and very high. Points associated with the healthy GDQS food groups increase for each higher quantity of consumption category. Points associated with the unhealthy GDQS food groups decrease for each higher quantity of consumption category. For the two food groups that are unhealthy in excessive consumption, points associated with the GDQS food group increase up to a threshold, then decrease. The overall hGDQS is the sum of the points across all 25 GDQS food groups. | GDQS has a range from 0 to 49.  Log transformed |
| Individual intakes | Caloric intake | Food consumption, in calories, on the previous day, with data based on 24 hour diet recall survey module | Log transformed |
|  | Calorie adequacy ratio (CAR) | Ratio of caloric intake to estimate average requirements (EAR) for calories, was used to determine the calorically adequacy of diets. A CAR value of 1 represents a calorically adequate diet. Estimated caloric requirements were calculated based on FAO guidelines and a dataset of Bangladeshi-specific requirements developed by Waid et al. Requirements were specific to an individual’s physical activity level as determined by primary occupation, pregnancy status, lactation status, and the ideal adult weight for each age and sex group given average physical stature of Bangladeshi adults (FAO, 2004; Picciano, 2003). | Log transformed |
|  | Global Diet Quality Score | Individual level GDQS based on 24 hour food intake data; see hGDQS for description of how this is calculated. | Log transformed |
| Empowerment | Empowerment score | The empowerment score is the weighted average of the 12 pro-WEAI indicators. | Ranges from 0 to 1 |
|  | Whether empowered | An individual is defined as empowered if s/he reaches the threshold of 75 percent or more of the weighted indicators. | =1 if yes, =0 otherwise |
|  | Attitudes score (9-45) | Respondents were asked about their agreement (1 “strongly disagree” to 5 “strongly agree) on nine statements related to attitudes and perceptions about themselves and other household members. Examples include:  I make important contributions to my family  I sometimes refrain from voicing my opinion because I fear being ignored/ridiculed  Women should stand up for themselves to get what they want  Husbands should help wives with household chores like cooking and taking care of children  We can change culture/tradition regarding what men/women do and how they relate to each other | Ranges from 9 to 45 |
|  | Gender parity | A household achieves gender parity if the woman respondent is empowered or her empowerment score is equal to or greater than that of the man respondent in the household. | =1 if yes, =0 otherwise |

**Table S3: Correlates of Attrition**

| VARIABLES |  |
| --- | --- |
| T(SAAO) | 0.011 |
|  | (0.009) |
| T(APK) | 0.013 |
|  | (0.008) |
| Experienced flooding | -0.130*** |
|  | (0.020) |
| Age, household head | 0.000 |
|  | (0.000) |
| Household head is female | -0.002 |
|  | (0.023) |
| Average grades school, men 18y or older | -0.001 |
|  | (0.001) |
| Average grades school, women 18y or older | -0.001 |
|  | (0.001) |
| Number of adults | 0.003 |
|  | (0.003) |
| Dependency ratio | 0.007 |
|  | (0.005) |
| Wealth index | 0.003* |
|  | (0.002) |
| Household has fishpond | 0.007 |
|  | (0.009) |
| Land operated (ha) | 0.001 |
|  | (0.003) |
| Mobile phones owned, number | -0.005 |
|  | (0.005) |
| Household owns television | -0.022** |
|  | (0.011) |
| Received extension visit related to crops | 0.013** |
|  | (0.006) |
| Received extension visit related to livestock, poultry, fish | 0.016 |
|  | (0.012) |
| Household has electricity | 0.011 |
|  | (0.009) |
| Constant | 1.077*** |
|  | (0.031) |
|  |  |
| Observations | 2,125 |
| R-squared | 0.043 |

Notes: Outcome variable equals one if household attrited, zero otherwise. Results estimated using a linear probability model. Standard errors adjusted for clustering at block level are in parentheses. *p<.10; **p<.05; ***p<.01. Sample size is 2,125. F statistic on joint significance of treatment covariates is 1.50 with a p-value of 0.23. Controls for location (upazila dummy variables) included but not reported.

**Table S4: Balance**

We have two treatment arms and 15 control variables. Given this, we estimate a multinomial logit where the base category is the control group and assess whether the estimated coefficients are jointly statistically significant. Note that as not all treatment groups are found in each upazila, upazila dummy variables are not included below. Only three variables are statistically significant and we do not reject the null that all control variables are jointly zero.

| Treatment Group | Variable | Coefficient | Standard Error |
| --- | --- | --- | --- |
| T(SAAO) |  |  |  |
|  | Experienced flooding | -0.456 | 0.56 |
|  | Age, household head | -0.009 | 0.01 |
|  | Household head is female | 0.500 | 0.51 |
|  | Average grades school, men 18y or older | 0.021 | 0.02 |
|  | Average grades school, women 18y or older | -0.014 | 0.03 |
|  | Number of adults | -0.005 | 0.07 |
|  | Dependency ratio | -0.051 | 0.12 |
|  | Wealth index | -0.050 | 0.08 |
|  | Household has fishpond | -0.295 | 0.29 |
|  | Land operated (ha) | 0.103 | 0.11 |
|  | Mobile phones owned, number | 0.156* | 0.08 |
|  | Household owns television | 0.018 | 0.20 |
|  | Received extension visit related to crops | 0.068 | 0.29 |
|  | Received extension visit related to livestock, poultry, fish | -0.819** | 0.41 |
|  | Household has electricity | -0.152 | 0.37 |
|  | Constant | 0.194 | 0.72 |
| T(APK) |  |  |  |
|  | Experienced flooding | -0.736 | 0.60 |
|  | Age, household head | -0.010 | 0.01 |
|  | Household head is female | 0.168 | 0.44 |
|  | Average grades school, men 18y or older | -0.001 | 0.02 |
|  | Average grades school, women 18y or older | 0.002 | 0.03 |
|  | Number of adults | -0.098 | 0.08 |
|  | Dependency ratio | -0.090 | 0.11 |
|  | Wealth index | 0.051 | 0.09 |
|  | Household has fishpond | -0.438 | 0.31 |
|  | Land operated (ha) | 0.092 | 0.12 |
|  | Mobile phones owned, number | 0.084 | 0.11 |
|  | Household owns television | -0.314 | 0.26 |
|  | Received extension visit related to crops | -0.024 | 0.23 |
|  | Received extension visit related to livestock, poultry, fish | -0.178 | 0.46 |
|  | Household has electricity | -0.735* | 0.40 |
|  | Constant | 1.424 | 0.86 |

Notes: Standard errors adjusted for clustering at block level are in parentheses. *p<.10; **p<.05; ***p<.01. Sample size is 2,069. F statistic on joint significance of all covariates is 1.30 with a p-value of 0.19.

**Table S5: Attendance at training sessions, by treatment arm**

|  | T(SAAO) | T(APK) | T(SAAO) = T(APK) |
| --- | --- | --- | --- |
|  | (N = 1274) | (N = 1254) | p-value |
| How many training sessions did you attend? |  |  |  |
| Mean (SD) | 14.68 (5.89) | 14.92 (4.84) | 0.29 |
| Median (Q1, Q3) | 17.0 (12.0, 19.0) | 16.0 (14.0, 18.0) |  |
| Females: Percentage of trainings attended |  |  |  |
| Mean (SD) | 82.07 (28.67) | 86.25 (18.86) | <0.01 |
| Median (Q1, Q3) | 89.5 (73.7, 100.0) | 89.5 (84.2, 100.0) |  |
| Males: Percentage of trainings attended |  |  |  |
| Mean (SD) | 72.14 (32.53) | 70.28 (28.86) | 0.32 |
| Median (Q1, Q3) | 84.2 (52.6, 100.0) | 78.9 (52.6, 89.5) |  |
| Did you attend training together with your spouse? (%) |  |  |  |
| Yes | 91.2 | 93.1 | 0.08 |
| Why did you not attend all trainings? (%) |  |  |  |
| Attended all sessions | 38.5 | 41.6 |  |
| Agricultural work | 6.6 | 8.2 |  |
| Work, other | 38.0 | 33.2 |  |
| Illness | 10.8 | 11.6 |  |
| Social obligation | 3.5 | 3.6 |  |
| Bad weather | 0.9 | 0.5 |  |
| Did not think the training would be useful | 1.7 | 1.3 | 0.19 |
| If you missed a session, did the ANGeL nutrition worker/SAAO come to you? (%) |  |  |  |
| Yes | 60.1 | 60.4 | 0.89 |
| If you missed a session, did another household member attend in your place? (%) |  |  |  |
| Yes | 7.8 | 17.0 | <0.01 |
| Was there dissatisfaction among your husband and/or in-laws of you attending? |  |  |  |
| Yes | 9.4 | 6.8 | 0.10 |
| If your husband refused to go, could you attend the training sessions alone? (%) |  |  |  |
| Yes | 46.0 | 53.3 | 0.01 |

**Table S6: Access and experience with training sessions**

|  | T(SAAO) | T(APK) | T(SAAO) = T(APK) |
| --- | --- | --- | --- |
|  | (N = 1274) | (N = 1254) | p-value |
| Distance (km) of the training venue from home (one way) |  |  |  |
| Mean (SD) | 0.54 (0.79) | 0.52 (0.58) | 0.55 |
| Median (Q1, Q3) | 0.2 (0.2, 0.5) | 0.5 (0.2, 0.6) |  |
| Distance (minute) of the training venue from home (one way) |  |  |  |
| Mean (SD) | 11.3 (12.46) | 10.8 (10.07) | 0.31 |
| Median (Q1, Q3) | 8.0 (5.0, 15.0) | 10.0 (5.0, 15.0) |  |
| How did you generally travel to the training? (%) |  |  | 0.12 |
| Walking | 95.1 | 96.1 |  |
| By rickshaw | 0.5 | 0.6 |  |
| By van/nosimon/korimon | 1.7 | 0.7 |  |
| By boat | 0.1 | 0.0 |  |
| Combination of ricshshaw/van/Boat or Other | 3.6 | 2.7 |  |
| What kind of difficulty did you face when coming to the training sessions? (%) |  |  | 0.06 |
| No difficulty | 90.5 | 93.1 |  |
| Rain | 5.3 | 4.9 |  |
| Vehicle was not available | 0.8 | 0.4 |  |
| Road condition was bad | 2.7 | 1.0 |  |
| Husband/Wife was not willing to come | 0.1 | 0.2 |  |
| Household members created obstacle, Other | 0.7 | 0.6 |  |
| Where did the training sessions take place? (%) |  |  | <0.01 |
| Inside a well ventilated closed room | 52.7 | 31.0 |  |
| Courtyard/open space | 45.6 | 67.9 |  |
| Inside a damp closed room, Other | 1.8 | 1.2 |  |
| How were the contents of the training sessions? (%) |  |  | <0.01 |
| Very informative | 80.3 | 86.2 |  |
| Moderately informative | 18.8 | 13.4 |  |
| Most of the contents were already known | 0.5 | 0.2 |  |
| Topics were difficult to understand, other | 0.4 | 0.3 |  |
| How did you like the way of delivery of the trainer? (%) |  |  | 0.03 |
| Very communicative and understable | 79.7 | 82.7 |  |
| Moderately communicative and understandable | 19.5 | 16.9 |  |
| Delivery was too fast to understand | 0.7 | 0.1 |  |
| Trainer was reading out the manual and was not explaining, other | 0.1 | 0.4 |  |
| Do you think the trainer was well prepared for the training? (%) |  |  | 0.49 |
| Trainer was very well prepared | 83.5 | 82.8 |  |
| Well-prepared | 15.2 | 16.2 |  |
| Moderately or not prepared | 1.3 | 1.1 |  |
| Did you always understand what was taught? (%) |  |  | 0.74 |
| Always | 54.2 | 51.5 |  |
| Mostly | 36.9 | 39.4 |  |
| Often | 8.1 | 8.4 |  |
| Seldom, never | 0.8 | 0.6 |  |
| When you did not understand the content, did you ask the trainer to repeat or explain? (%) |  |  |  |
| Yes | 90.7 | 92.1 | 0.24 |
| When you asked the trainer to explain again, how did s/he react? (%) |  |  |  |
| S/he repeated happily | 94.2 | 97.5 | <0.01 |
| Who did you discuss/share information from the training sessions with? (%) |  |  | . |
| Spouse | 95.6 | 96.5 | 0.30 |
| Fellow Trainees | 94.7 | 96.5 | 0.04 |
| Other household members or relatives | 94.2 | 95.5 | 0.17 |
| Neighbors | 85.0 | 85.2 | 0.89 |
| Friends | 46.4 | 45.2 | 0.58 |
| Community members | 7.0 | 10.6 | <0.01 |
| Other | 0.2 | 0.3 | 0.64 |
| Did not discuss/share with anyone | 1.6 | 1.1 | 0.28 |

**Table S7: Women’s perception of training sessions**

|  | T(SAAO) | T(APK) | T(SAAO) = T(APK) |
| --- | --- | --- | --- |
|  | (N = 624) | (N = 614) | p-value |
| Were the training sessions helpful? |  |  |  |
| Yes very helpful | 97.6 | 99.5 | 0.02 |
| If yes, how were the trainings helpful? |  |  |  |
| Post-training increase in income | 12.5 | 6.8 | <0.01 |
| Learnt new agricultural practices | 23.9 | 8.6 | <0.01 |
| Care of children and nutrition | 84.5 | 86.5 | 0.34 |
| Maternal care and nutrition | 69.6 | 73.4 | 0.16 |
| Intra-household relationship improved | 5.1 | 5.8 | 0.61 |
| Household health status improved | 12.7 | 16.6 | 0.06 |
| Children’s health improved | 19.2 | 21.2 | 0.39 |
| Do you feel that you have gained more respect/status within your household? |  |  | 0.01 |
| Yes | 74.6 | 81.9 |  |
| No | 7.0 | 5.2 |  |
| No, because I have always been respected | 18.4 | 12.9 |  |
| Do you feel more confident in making decisions about spending money? |  |  | 0.02 |
| Yes | 77.7 | 83.4 |  |
| No, I do not feel more confident | 4.5 | 4.5 |  |
| No, because I had enough confidence before | 17.8 | 12.0 |  |
| Do you feel that you have gained more respect within the community? |  |  | 0.34 |
| Yes | 71.9 | 75.3 |  |
| No | 11.8 | 11.3 |  |
| No, because I have always been respected | 16.3 | 13.4 |  |
| Did the group participation result in solidarity/close ties among participants? |  |  | <0.01 |
| Yes | 89.5 | 95.5 |  |
| Did participation in the program interfere with your domestic responsibilities? |  |  | 0.02 |
| Yes | 30.9 | 24.7 |  |
| Do you meet with any new friends after training? (% Yes) | 87.0 | 91.5 | 0.01 |

**Table S8: Men’s perception of training sessions**

|  | T(SAAO) | T(APK) | T(SAAO) = T(APK) |
| --- | --- | --- | --- |
|  | (N = 637) | (N = 627) | p-value |
| Were the training sessions helpful? |  |  | 0.88 |
| Yes very helpful | 91.3 | 92.1 |  |
| If yes, how were the trainings helpful? |  |  |  |
| Post-training increase in income | 25.9 | 16.9 | <0.01 |
| Learnt new agricultural practices | 52.6 | 34.1 | <0.01 |
| Care of children and nutrition | 72.7 | 81.9 | <0.01 |
| Maternal care and nutrition | 40.7 | 43.7 | 0.33 |
| Intra-household relationship improved | 4.0 | 5.3 | 0.35 |
| Household health status improved | 9.3 | 9.8 | 0.81 |
| Children’s health improved | 6.3 | 6.1 | 0.90 |
| Do you feel that you have gained more respect/ status within your house? |  |  | 0.16 |
| Yes | 75.0 | 70.0 |  |
| No | 8.5 | 9.4 |  |
| No, because I have always been respected | 16.5 | 20.6 |  |
| Do you feel more confident in making decisions about spending money? |  |  | 0.76 |
| Yes | 75.6 | 74.2 |  |
| No, I do not feel more confident | 7.6 | 7.3 |  |
| No, because I had enough confidence before | 16.8 | 18.5 |  |
| Do you feel that you have gained more respect within the community? |  |  | 0.14 |
| Yes | 72.3 | 68.0 |  |
| No | 11.3 | 10.9 |  |
| No, because I have always been respected | 16.5 | 21.2 |  |
| Did the group participation result in solidarity/close ties among participants? |  |  | 0.17 |
| Yes | 85.8 | 88.6 |  |
| Does participation in the program interfere with your domestic responsibilities? |  |  | 0.38 |
| Yes | 51.4 | 48.7 |  |
| Do you meet with any new friends after training? (% Yes) | 78.2 | 81.3 | 0.21 |

**Table S9: Knowledge and adoption of improved agricultural production practices:** **crops, livestock, fish. By sex**

**WOMEN**

|  | (1) | (2) | (3) |  | (4) | (5) | (6) |
| --- | --- | --- | --- | --- | --- | --- | --- |
|  | Score on test of knowledge of improved: | | |  | Any adoption, improved | | |
|  | Agricultural practices | Livestock practices | Fishpond practices |  | Agricultural practices | Livestock practices | Fishpond practices |
| Treatments |  |  |  |  |  |  |  |
| T(SAAO) | 0.869*** | 0.594*** | 0.038 |  | 0.090*** | 0.070** | 0.010 |
|  | (0.175) | (0.202) | (0.071) |  | (0.031) | (0.030) | (0.017) |
| T(APK) | 0.583*** | 0.427** | 0.085 |  | 0.049* | 0.062*** | -0.000 |
|  | (0.129) | (0.200) | (0.059) |  | (0.027) | (0.023) | (0.013) |
| P values on equality of treatments |  |  |  |  |  |  |  |
| T(SAAO) = T(APK) | 0.09 | 0.39 | 0.56 |  | 0.22 | 0.78 | 0.56 |
|  |  |  |  |  |  |  |  |
| Mean, control group | 5.4 | 8.9 | 2.0 |  | 0.16 | 0.17 | 0.11 |
| Observations | 2,061 | 2,069 | 2,061 |  | 2,061 | 2,061 | 2,061 |
| R-squared | 0.234 | 0.271 | 0.187 |  | 0.152 | 0.238 | 0.117 |

**MEN**

|  | (1) | (2) | (3) |  | (4) | (5) | (6) |
| --- | --- | --- | --- | --- | --- | --- | --- |
|  | Score on test of knowledge of improved: | | |  | Any adoption, improved | | |
|  | Agricultural practices | Livestock practices | Fishpond practices |  | Agricultural practices | Livestock practices | Fishpond practices |
| Treatments |  |  |  |  |  |  |  |
| T(SAAO) | 1.017*** | 1.259*** | 0.076* |  | 0.191*** | 0.138*** | 0.095*** |
|  | (0.174) | (0.202) | (0.043) |  | (0.031) | (0.025) | (0.021) |
| T(APK) | 0.863*** | 0.998*** | 0.159*** |  | 0.158*** | 0.123*** | 0.050** |
|  | (0.169) | (0.185) | (0.043) |  | (0.032) | (0.031) | (0.020) |
| P values on equality of treatments |  |  |  |  |  |  |  |
| T(SAAO) = T(APK) | 0.42 | 0.15 | 0.09 |  | 0.36 | 0.64 | 0.03 |
|  |  |  |  |  |  |  |  |
| Mean, control group | 5.8 | 8.0 | 2.5 |  | 0.12 | 0.09 | 0.11 |
| Observations | 1,929 | 1,929 | 1,929 |  | 1,929 | 1,929 | 1,929 |
| R-squared | 0.209 | 0.198 | 0.184 |  | 0.182 | 0.142 | 0.178 |

Note: Estimates are intent-to-treat. Standard errors adjusted for clustering at block level are in parentheses. *p<.10; **p<.05; ***p<.01. All specifications include as independent variables the treatment indicators and the control variables listed in the notes to Table 1. Controlling for the familywise error rate (FWER) using the method described by Romano and Wolf (2005) does not alter the pattern of statistical significance described here.

**Table S10: Single-difference impacts on attitudes statements**

|  |  | Impacts |  | Test of difference between arms |
| --- | --- | --- | --- | --- |
| Indicator | Control | T(SAAO) | T(APK) | T(SAAO) = T(APK) |
|  | Mean |  |  | p-value |
|  | (SE) |  |  |  |
| *WOMEN* |  |  |  |  |
| I make important contributions to my family | 4.68 | 0.05 | 0.05 | 0.95 |
|  | (0.03) | (0.03) | (0.03) |  |
| Some adults in my family do not make important contributions to the family | 2.40 | -0.08 | -0.12 | 0.66 |
|  | (0.09) | (0.10) | (0.10) |  |
| I make important contributions to my community | 3.23 | 0.13** | 0.16** | 0.62 |
|  | (0.07) | (0.06) | (0.07) |  |
| I sometimes refrain from voicing my opinion because I fear being ignored/ridiculed | 3.46 | 0.03 | -0.16* | 0.04** |
|  | (0.08) | (0.06) | (0.09) |  |
| I have a hard time saying positive things about myself | 3.51 | -0.01 | -0.14 | 0.14 |
|  | (0.07) | (0.08) | (0.09) |  |
| Women should stand up for themselves to get what they want | 4.67 | 0.07* | 0.05 | 0.68 |
|  | (0.03) | (0.04) | (0.04) |  |
| Women are usually very busy with work that benefits the household | 4.74 | -0.02 | 0.01 | 0.41 |
|  | (0.03) | (0.04) | (0.04) |  |
| Husbands should help wives with household chores like cooking and taking care of children | 4.62 | 0.05 | 0.05 | 0.93 |
|  | (0.04) | (0.05) | (0.04) |  |
| We can change culture/tradition regarding what men/women do and how they relate | 3.87 | 0.09 | -0.01 | 0.18 |
|  | (0.10) | (0.06) | (0.06) |  |
| I make important contributions to my family | 4.60 | 0.07** | 0.03 | 0.25 |
|  | (0.04) | (0.03) | (0.03) |  |
| Some adults in my family do not make important contributions to the family | 2.46 | -0.11 | -0.02 | 0.41 |
|  | (0.09) | (0.10) | (0.08) |  |
| *MEN* |  |  |  |  |
| I make important contributions to my community | 3.77 | 0.11** | -0.05 | 0.01** |
|  | (0.06) | (0.05) | (0.06) |  |
| I sometimes refrain from voicing my opinion because I fear being ignored/ridiculed | 3.19 | -0.03 | -0.03 | 0.98 |
|  | (0.08) | (0.08) | (0.07) |  |
| I have a hard time saying positive things about myself | 3.36 | 0.01 | -0.02 | 0.68 |
|  | (0.08) | (0.08) | (0.08) |  |
| Women should stand up for themselves to get what they want | 4.45 | -0.01 | -0.07 | 0.28 |
|  | (0.05) | (0.05) | (0.06) |  |
| Women are usually very busy with work that benefits the household | 4.51 | 0.04 | -0.06 | 0.03** |
|  | (0.04) | (0.04) | (0.05) |  |
| Husbands should help wives with household chores like cooking and taking care of children | 4.42 | 0.08* | -0.00 | 0.09* |
|  | (0.04) | (0.04) | (0.04) |  |
| We can change culture/tradition regarding what men/women do and how they relate | 3.74 | 0.17** | 0.05 | 0.10* |
|  | (0.07) | (0.07) | (0.07) |  |

Note: Sample size is 1,737.

**Table S11:** **Assessing robustness to exclusion of control variables**

**Table S11A: Nutrition, agricultural knowledge and adoption of improved practices: Women**

|  | (1) | (2) | (3) | (4) |  | (5) | (6) | (7) | (8) |
| --- | --- | --- | --- | --- | --- | --- | --- | --- | --- |
|  | Nutrition knowledge, percent correct | Agriculture knowledge, percent correct | Any adoption, improved agricultural practices | Number, improved agricultural practices |  | Nutrition knowledge, percent correct | Agriculture knowledge, percent correct | Any adoption, improved agricultural practices | Number, improved agricultural practices |
|  | No controls | | | |  | Controls included | | | |
| Treatments |  |  |  |  |  |  |  |  |  |
| T(SAAO) | 3.484*** | 5.768** | 0.143** | 0.721** |  | 3.257*** | 4.608*** | 0.094*** | 0.502*** |
|  | (1.021) | (2.243) | (0.068) | (0.313) |  | (0.663) | (1.201) | (0.031) | (0.159) |
| T(APK) | 3.802*** | 1.212 | 0.035 | 0.040 |  | 4.162*** | 3.434*** | 0.075*** | 0.274** |
|  | (0.878) | (1.789) | (0.053) | (0.216) |  | (0.671) | (1.007) | (0.026) | (0.112) |
|  |  |  |  |  |  |  |  |  |  |
| Observations | 2,060 | 2,069 | 2,061 | 2,061 |  | 2,060 | 2,069 | 2,061 | 2,061 |
| R-squared | 0.038 | 0.029 | 0.017 | 0.026 |  | 0.167 | 0.266 | 0.223 | 0.233 |

Notes: Estimates are intent-to-treat from OLS models. Standard errors adjusted for clustering at block level are in parentheses. *p<.10; **p<.05; ***p<.01. No controls includes only treatment status. “Controls included” include as independent variables the treatment indicators, baseline values for the outcome variable (except for those outcomes relating to agricultural knowledge and practice) and the following control variables: age and sex of household head, mean education levels of males and females 18 and older, number of adults, dependency ratio, wealth index, land owned at baseline, fishpond owned at baseline, baseline access to information as measured by (baseline) number of mobile phones owned, ownership of television, received extension visit for crop production, received extension visit for livestock or fish production, household has access to electricity, and baseline upazila.

**Table S11B: Nutrition, agricultural knowledge and adoption of improved practices: Men**

|  | (1) | (2) | (3) | (4) |  | (5) | (6) | (7) | (8) |
| --- | --- | --- | --- | --- | --- | --- | --- | --- | --- |
|  | Nutrition knowledge, percent correct | Agriculture knowledge, percent correct | Any adoption, improved agricultural practices | Number, improved agricultural practices |  | Nutrition knowledge, percent correct | Agriculture knowledge, percent correct | Any adoption, improved agricultural practices | Number, improved agricultural practices |
|  | No controls | | | |  | Controls included | | | |
| Treatments |  |  |  |  |  |  |  |  |  |
| T(SAAO) | 5.814*** | 9.018*** | 0.270*** | 1.087*** |  | 4.894*** | 7.351*** | 0.244*** | 0.868*** |
|  | (1.165) | (2.008) | (0.052) | (0.260) |  | (0.698) | (1.078) | (0.037) | (0.157) |
| T(APK) | 3.780*** | 4.664** | 0.157*** | 0.555** |  | 4.240*** | 6.312*** | 0.182*** | 0.711*** |
|  | (1.334) | (1.929) | (0.056) | (0.229) |  | (0.781) | (0.973) | (0.037) | (0.158) |
|  |  |  |  |  |  |  |  |  |  |
| Observations | 1,738 | 1,929 | 1,929 | 1,929 |  | 1,638 | 1,929 | 1,929 | 1,929 |
| R-squared | 0.039 | 0.057 | 0.059 | 0.042 |  | 0.198 | 0.255 | 0.199 | 0.189 |

Notes: See Table S11A.

**Table S11C: Field crops**

|  | (1) | (2) |  | (3) | (4) |
| --- | --- | --- | --- | --- | --- |
|  | No controls | |  | Controls included | |
|  | Simpson Diversification Index | Number, non-rice field crops |  | Simpson Diversification Index | Number, non-rice field crops |
| Treatments |  |  |  |  |  |
| T(SAAO) | -0.025 | -0.100 |  | 0.006 | 0.035 |
|  | (0.037) | (0.131) |  | (0.019) | (0.082) |
| T(APK) | 0.014 | 0.083 |  | 0.007 | 0.041 |
|  | (0.043) | (0.165) |  | (0.020) | (0.091) |
|  |  |  |  |  |  |
| Observations | 1,825 | 2,069 |  | 1,825 | 2,069 |
| R-squared | 0.004 | 0.003 |  | 0.397 | 0.272 |

Notes: See Table S11A.

**Table S11D: Homestead agricultural production diversification**

|  | (1) | (2) | (3) | (4) |  | (5) | (6) | (7) | (8) |
| --- | --- | --- | --- | --- | --- | --- | --- | --- | --- |
|  | No controls | | | |  | Controls included | | | |
|  | Number, homestead garden crops | Any egg production | Any dairy production | Any fish production |  | Number, homestead garden crops | Any egg production | Any dairy production | Any fish production |
| Treatments |  |  |  |  |  |  |  |  |  |
| T(SAAO) | 0.061 | 0.046 | 0.020 | 0.014 |  | 0.005 | 0.034 | 0.023 | 0.004 |
|  | (0.222) | (0.033) | (0.031) | (0.052) |  | (0.125) | (0.026) | (0.020) | (0.030) |
| T(APK) | 0.336 | 0.053* | 0.045 | -0.011 |  | 0.151 | 0.068*** | 0.062** | -0.001 |
|  | (0.277) | (0.030) | (0.037) | (0.052) |  |  |  |  |  |
|  |  |  |  |  |  |  |  |  |  |
| Observations | 2,069 | 2,069 | 2,069 | 2,069 |  | 2,069 | 2,069 | 2,069 | 2,069 |
| R-squared | 0.007 | 0.003 | 0.002 | <0.001 |  | 0.294 | 0.118 | 0.197 | 0.207 |

Notes: See Table S11A

**Table S11E: Homestead production and consumption**

**No controls**

|  | (1) | (2) | (3) | (4) |  | (5) | (6) | (7) | (8) |
| --- | --- | --- | --- | --- | --- | --- | --- | --- | --- |
|  | Production | | | |  | Consumption | | | |
|  | Fruit and vegetables | Eggs | Dairy | Fish |  | Fruit and vegetables | Eggs | Dairy | Fish |
| Treatments |  |  |  |  |  |  |  |  |  |
| T(SAAO) | 0.162 | 0.220 | 0.100 | 0.170 |  | 0.160 | 0.250 | 0.123 | 0.155 |
|  | (0.190) | (0.186) | (0.183) | (0.258) |  | (0.180) | (0.182) | (0.158) | (0.233) |
| T(APK) | 0.138 | 0.255 | 0.196 | 0.021 |  | 0.076 | 0.202 | 0.220 | 0.031 |
|  | (0.246) | (0.173) | (0.205) | (0.263) |  | (0.217) | (0.165) | (0.181) | (0.237) |
|  |  |  |  |  |  |  |  |  |  |
| Observations | 2,069 | 2,069 | 2,069 | 2,069 |  | 2,069 | 2,069 | 2,069 | 2,069 |
| R-squared | 0.002 | 0.003 | 0.001 | 0.001 |  | 0.002 | 0.003 | 0.002 | 0.001 |

**Controls included**

|  | (1) | (2) | (3) | (4) |  | (5) | (6) | (7) | (8) |
| --- | --- | --- | --- | --- | --- | --- | --- | --- | --- |
|  | Production | | | |  | Consumption | | | |
|  | Fruit and vegetables | Eggs | Dairy | Fish |  | Fruit and vegetables | Eggs | Dairy | Fish |
| Treatments |  |  |  |  |  |  |  |  |  |
| T(SAAO) | -0.058 | 0.169 | 0.139 | 0.080 |  | -0.033 | 0.187 | 0.146 | 0.050 |
|  | (0.102) | (0.139) | (0.109) | (0.125) |  | (0.091) | (0.123) | (0.100) | (0.118) |
|  |  |  |  |  |  |  |  |  |  |
| T(APK) | 0.110 | 0.409*** | 0.320** | 0.136 |  | 0.106 | 0.385*** | 0.311** | 0.105 |
|  | (0.100) | (0.132) | (0.130) | (0.105) |  | (0.097) | (0.114) | (0.123) | (0.095) |
|  |  |  |  |  |  |  |  |  |  |
| Observations | 2,069 | 2,069 | 2,069 | 2,069 |  | 2,069 | 2,069 | 2,069 | 2,069 |
| R-squared | 0.277 | 0.147 | 0.211 | 0.311 |  | 0.273 | 0.148 | 0.203 | 0.274 |

Notes: See Table S11A

**Table S11F: Household diet**

|  | (1) | (2) | (3) |  | (4) | (5) | (6) |
| --- | --- | --- | --- | --- | --- | --- | --- |
|  | No controls | | |  | Controls included | | |
|  | Dietary Diversity Score | Log per capita caloric acquisition | Log household Global Diet Quality Score |  | Dietary Diversity Score | Log per capita caloric acquisition | Log household Global Diet Quality Score |
| Treatments |  |  |  |  |  |  |  |
| T-N | 0.179 | 0.039* | 0.068*** |  | 0.163** | 0.028* | 0.061*** |
|  | (0.194) | (0.023) | (0.023) |  | (0.080) | (0.016) | (0.010) |
| T-A | 0.247 | 0.027 | 0.034 |  | 0.332*** | 0.020 | 0.050*** |
|  | (0.194) | (0.021) | (0.024) |  | (0.095) | (0.015) | (0.012) |
|  |  |  |  |  |  |  |  |
| Observations | 2,069 | 2,074 | 2,074 |  | 2,069 | 2,069 | 2,069 |
| R-squared | 0.006 | 0.004 | 0.024 |  | 0.271 | 0.109 | 0.285 |

Notes: See Table S11A.

**Table S11G: Individual intakes**

**All individuals**

|  | (1) | (2) | (3) |  | (4) | (5) | (6) |
| --- | --- | --- | --- | --- | --- | --- | --- |
|  | No controls | | |  | Controls included | | |
|  | Log caloric intake | Log calorie adequacy ratio | Log Global Diet Quality Score |  | Log caloric intake | Log calorie adequacy ratio | Log Global Diet Quality Score |
| Treatments |  |  |  |  |  |  |  |
| T-SAAO | 0.010 | -0.009 | 0.083* |  | 0.003 | -0.001 | 0.055** |
|  | (0.024) | (0.008) | (0.042) |  | (0.015) | (0.006) | (0.021) |
|  |  |  |  |  |  |  |  |
| T-APK | 0.013 | 0.0001 | 0.052 |  | 0.002 | -0.001 | 0.090*** |
|  |  |  |  |  |  |  |  |
|  | (0.022) | (0.009) | (0.039) |  | (0.013) | (0.006) | (0.020) |
|  |  |  |  |  |  |  |  |
| Observations | 5,490 | 5,490 | 5,490 |  | 5,490 | 5,490 | 5,490 |
| R-squared | 0.057 | 0.73 | 0.061 |  | 0.19 | 0.77 | 0.21 |

Notes: See Table S11A.

**Males**

|  | (1) | (2) | (3) |  | (4) | (5) | (6) |
| --- | --- | --- | --- | --- | --- | --- | --- |
|  | No controls | | |  | Controls included | | |
|  | Log caloric intake | Log calorie adequacy ratio | Log Global Diet Quality Score |  | Log caloric intake | Log calorie adequacy ratio | Log Global Diet Quality Score |
| Treatments |  |  |  |  |  |  |  |
| T-SAAO | 0.014 | 0.013* | 0.080* |  | 0.008 | 0.012 | 0.055** |
|  | (0.029) | (0.007) | (0.044) |  | (0.021) | (0.007) | (0.021) |
|  |  |  |  |  |  |  |  |
| T-APK | 0.013 | 0.008 | 0.058 |  | -0.004 | 0.008 | 0.090*** |
|  | (0.026) | (0.006) | (0.041) |  | (0.016) | (0.006) | (0.022) |
|  |  |  |  |  |  |  |  |
| Observations | 2,501 | 2,501 | 2,501 |  | 2,501 | 2,501 | 2,501 |
| R-squared | 0.046 | 0.78 | 0.056 |  | 0.16 | 0.78 | 0.21 |

**Females**

|  | (1) | (2) | (3) |  | (4) | (5) | (6) |
| --- | --- | --- | --- | --- | --- | --- | --- |
|  | No controls | | |  | Controls included | | |
|  | Log caloric intake | Log calorie adequacy ratio | Log Global Diet Quality Score |  | Log caloric intake | Log calorie adequacy ratio | Log Global Diet Quality Score |
| Treatments |  |  |  |  |  |  |  |
| T-SAAO | .0071 | -0.020 | 0.087* |  | -0.003 | -0.007 | 0.055** |
|  | (0.022) | (0.013) | (0.042) |  | (0.014) | (0.009) | (0.023) |
|  |  |  |  |  |  |  |  |
| T-APK | .015 | -0.004 | 0.046 |  | 0.005 | -0.003 | 0.088*** |
|  | (0.021) | (0.015) | (0.038) |  | (0.013) | (0.010) | (0.021) |
|  |  |  |  |  |  |  |  |
| Observations | 2,989 | 2,989 | 2,989 |  | 2,989 | 2,989 | 2,989 |
| R-squared | 0.038 | 0.72 | 0.065 |  | 0.15 | 0.75 | 0.21 |

**Table S11H: Empowerment**

**Women**

|  | (1) | (2) | (3) |  | (4) | (5) | (6) |
| --- | --- | --- | --- | --- | --- | --- | --- |
|  | No controls | | |  | Controls included | | |
|  | Empowerment score | Whether empowered | Total gender attitudes score |  | Empowerment score | Whether empowered | Total gender attitudes score |
| Treatments |  |  |  |  |  |  |  |
| T(SAAO) | 0.042*** | 0.072** | 0.360 |  | 0.035*** | 0.053** | 0.433 |
|  | (0.016) | (0.032) | (0.463) |  | (0.011) | (0.026) | (0.265) |
| T(APK) | 0.040** | 0.073* | 0.971** |  | 0.034*** | 0.066** | 0.721** |
|  | (0.016) | (0.037) | (0.449) |  | (0.011) | (0.029) | (0.298) |
|  |  |  |  |  |  |  |  |
| Observations | 1,743 | 1,743 | 1,743 |  | 1,743 | 1,743 | 1,743 |
| R-squared | 0.015 | 0.006 | 0.010 |  | 0.123 | 0.068 | 0.151 |

**Men**

|  | (1) | (2) | (3) |  | (4) | (5) | (6) | (7) | (8) |
| --- | --- | --- | --- | --- | --- | --- | --- | --- | --- |
|  | No controls | | |  | Controls included | | | No controls | Controls included |
|  | Empowerment score | Whether empowered | Total gender attitudes score |  | Empowerment score | Whether empowered | Total gender attitudes score | Gender parity | |
| Treatments |  |  |  |  |  |  |  |  |  |
| T(SAAO) | 0.030** | 0.096** | 0.542 |  | 0.031*** | 0.089*** | 0.609** | 0.046 | 0.020 |
|  | (0.014) | (0.044) | (0.333) |  | (0.010) | (0.032) | (0.248) | (0.041) | (0.029) |
| T(APK) | 0.036** | 0.113** | 0.300 |  | 0.027*** | 0.084*** | -0.010 | 0.027 | 0.018 |
|  | (0.015) | (0.044) | (0.335) |  | (0.009) | (0.031) | (0.195) | (0.040) | (0.032) |
|  |  |  |  |  |  |  |  |  |  |
| Observations | 1,743 | 1,743 | 1,743 |  | 1,743 | 1,743 | 1,743 | 1,743 | 1,743 |
| R-squared | 0.014 | 0.011 | 0.003 |  | 0.131 | 0.089 | 0.082 | 0.002 | 0.082 |

Notes: See Table S11A. Estimates are single difference. Sample is restricted to households where both women and men complete the survey modules needed to construct the Pro-WEAI.

**Table S12: Adjusting P values for multiple hypothesis tests**

**Table S12A: Nutrition, agricultural knowledge, and adoption of improved practices: Women**

|  | Domain | Outcome | P Value | Treatment |  |
| --- | --- | --- | --- | --- | --- |
|  |  |  |  | T(SAAO) | T(APK) |
| (1) | Nutrition and Agriculture Knowledge | Nutrition knowledge, percent correct | Regression P value | <0.0001 | <0.0001 |
|  |  |  | Romano-Wolf P value | 0.0010 | 0.0010 |
|  |  | Agriculture knowledge, percent correct | Regression P value | 0.0003 | 0.0011 |
|  |  |  | Romano-Wolf P value | 0.0010 | 0.0010 |
|  |  |  |  |  |  |
| (2) | Any adoption | Any adoption, improved agricultural practices | Regression P value | 0.0030 | 0.0051 |
|  |  |  | Romano-Wolf P value | 0.0010 | 0.0010 |
|  |  |  |  |  |  |
| (3) | Number of adopted practices | Number, improved agricultural practices | Regression P value | 0.0023 | 0.0176 |
|  |  |  | Romano-Wolf P value | 0.0010 | 0.0010 |

**Table S12B: Nutrition, agricultural knowledge, and adoption of improved practices: Men**

|  | Domain | Outcome | P Value | Treatment |  |
| --- | --- | --- | --- | --- | --- |
|  |  |  |  | T(SAAO) | T(APK) |
| (1) | Nutrition and Agriculture Knowledge | Nutrition knowledge, percent correct | Regression P value | <0.0001 | <0.0001 |
|  |  |  | Romano-Wolf P value | 0.0010 | 0.0010 |
|  |  | Agriculture knowledge, percent correct | Regression P value | <0.0001 | <0.0001 |
|  |  |  | Romano-Wolf P value | 0.0010 | 0.0010 |
|  |  |  |  |  |  |
| (2) | Any adoption | Any adoption, improved agricultural practices | Regression P value | <0.0001 | <0.0001 |
|  |  |  | Romano-Wolf P value | 0.0010 | 0.0010 |
|  |  |  |  |  |  |
| (3) | Number of adopted practices | Number, improved agricultural practices | Regression P value | <0.0001 | <0.0001 |
|  |  |  | Romano-Wolf P value | 0.0010 | 0.0010 |

**Table S12C: Field crops and homestead agricultural production diversification**

|  | Domain | Outcome | P Value | Treatment |  |
| --- | --- | --- | --- | --- | --- |
|  |  |  |  | T(SAAO) | T(APK) |
| (1) | Diversification of field crops | Simpson Diversification Index | Regression P value | 0.7458 | 0.7202 |
|  |  |  | Romano-Wolf P value | 0.8312 | 0.8312 |
|  |  | Number, non-rice field crops | Regression P value | 0.6714 | 0.6598 |
|  |  |  | Romano-Wolf P value | 0.8312 | 0.8312 |
|  |  |  |  |  |  |
| (2) | Diversification of homestead food production | Number, homestead garden crops | Regression P value | 0.9713 | 0.1496 |
|  |  |  | Romano-Wolf P value | 0.9990 | 0.2577 |
|  |  | Any egg production | Regression P value | 0.2048 | 0.0102 |
|  |  |  | Romano-Wolf P value | 0.3417 | 0.0100 |
|  |  | Any dairy production | Regression P value | 0.2494 | 0.0114 |
|  |  |  | Romano-Wolf P value | 0.3716 | 0.0110 |
|  |  | Any fish production | Regression P value | 0.9055 | 0.9764 |
|  |  |  | Romano-Wolf P value | 0.9960 | 0.9960 |

**Table S12D: Homestead production and consumption**

|  | Domain | Outcome | P Value | Treatment |  |
| --- | --- | --- | --- | --- | --- |
|  |  |  |  | T(SAAO) | T(APK) |
| (1) | Production | Fruit and vegetables | Regression P value | 0.5730 | 0.2797 |
|  |  |  | Romano-Wolf P value | 0.5734 | 0.4156 |
|  |  | Eggs | Regression P value | 0.2305 | 0.0028 |
|  |  |  | Romano-Wolf P value | 0.4156 | 0.0020 |
|  |  | Dairy | Regression P value | 0.2068 | 0.0171 |
|  |  |  | Romano-Wolf P value | 0.4156 | 0.0190 |
|  |  | Fish | Regression P value | 0.5254 | 0.2008 |
|  |  |  | Romano-Wolf P value | 0.5734 | 0.4156 |
|  |  |  |  |  |  |
| (2) | Consumption | Fruit and vegetables | Regression P value | 0.7195 | 0.2813 |
|  |  |  | Romano-Wolf P value | 0.7902 | 0.4116 |
|  |  | Eggs | Regression P value | 0.1357 | 0.0012 |
|  |  |  | Romano-Wolf P value | 0.2358 | 0.0010 |
|  |  | Dairy | Regression P value | 0.1497 | 0.2358 |
|  |  |  | Romano-Wolf P value | 0.0137 | 0.0150 |
|  |  | Fish | Regression P value | 0.6743 | 0.2773 |
|  |  |  | Romano-Wolf P value | 0.7902 | 0.4116 |

**Table S12E: Household diet**

|  | Domain | Outcome | P Value | Treatment |  |
| --- | --- | --- | --- | --- | --- |
|  |  |  |  | T-N | T-A |
| (1) | Household diet | Dietary Diversity Score | Regression P value | 0.0454 | 0.0009 |
|  |  |  | Romano-Wolf P value | 0.0210 | 0.0010 |
|  |  | Log per capita caloric acquisition | Regression P value | 0.0894 | 0.1837 |
|  |  |  | Romano-Wolf P value | 0.0480 | 0.0819 |
|  |  | Log household Global Diet Quality Score | Regression P value | <0.0001 | 0.0010 |
|  |  |  | Romano-Wolf P value | 0.0001 | 0.0010 |

**Table S12F: Individual diets**

|  | Domain | Outcome | P Value | Treatment |  |
| --- | --- | --- | --- | --- | --- |
|  |  |  |  | T-SAAO | T-APK |
| (2) | All individuals, ages 15+ | Log caloric intake | Regression P value | 0.8613 | 0.8856 |
|  |  |  | Romano-Wolf P value | 0.9321 | 0.9321 |
|  |  | Log calorie adequacy ratio | Regression P value | 0.8740 | 0.8414 |
|  |  |  | Romano-Wolf P value | 0.9451 | 0.9451 |
|  |  | Log Global Diet Quality Score | Regression P value | 0.0125 | 0.0001 |
|  |  |  | Romano-Wolf P value | 0.0010 | 0.0010 |
|  |  |  |  |  |  |
| (2) | Males, ages 15+ | Log caloric intake | Regression P value | 0.7180 | 0.8104 |
|  |  |  | Romano-Wolf P value | 0.8102 | 0.8102 |
|  |  | Log calorie adequacy ratio | Regression P value | 0.0895 | 0.2182 |
|  |  |  | Romano-Wolf P value | 0.0909 | 0.1768 |
|  |  | Log Global Diet Quality Score | Regression P value | 0.0146 | 0.0002 |
|  |  |  | Romano-Wolf P value | 0.0020 | 0.0010 |
|  |  |  |  |  |  |
| (3) | Females, ages 15+ | Log caloric intake | Regression P value | 0.8024 | 0.6727 |
|  |  |  | Romano-Wolf P value | 0.7962 | 0.7962 |
|  |  | Log calorie adequacy ratio | Regression P value | 0.4257 | 0.7554 |
|  |  |  | Romano-Wolf P value | 0.3696 | 0.6144 |
|  |  | Log Global Diet Quality Score | Regression P value | 0.0210 | 0.0002 |
|  |  |  | Romano-Wolf P value | 0.0020 | 0.0010 |

**Table S12G: Empowerment**

|  | Domain | Outcome | P Value | Treatment | |
| --- | --- | --- | --- | --- | --- |
|  |  |  |  | T(SAAO) | T(APK) |
| (1) | Women | Empowerment score | Regression P value | 0.0028 | 0.0039 |
|  |  |  | Romano-Wolf P value | 0.0010 | 0.0030 |
|  |  | Whether empowered | Regression P value | 0.0451 | 0.0265 |
|  |  |  | Romano-Wolf P value | 0.0180 | 0.0110 |
|  |  | Total gender attitudes score | Regression P value | 0.1084 | 0.0186 |
|  |  |  | Romano-Wolf P value | 0.0230 | 0.0090 |
|  |  |  |  |  |  |
| (2) | Men | Empowerment score | Regression P value | 0.0031 | 0.0063 |
|  |  |  | Romano-Wolf P value | 0.0010 | 0.0010 |
|  |  | Whether empowered | Regression P value | 0.0077 | 0.0095 |
|  |  |  | Romano-Wolf P value | 0.0010 | 0.0010 |
|  |  | Total gender attitudes score | Regression P value | 0.0169 | 0.9608 |
|  |  |  | Romano-Wolf P value | 0.0040 | 0.9530 |

**Table S13: Impacts on measures of gender attitudes statements, by presence of mother-in-law**

|  | Mother-in-law not present | | Mother-in-law present | | Test of with Mother-in-law present vs. Mother-in-law not present | Test of with Mother-in-law present vs. Mother-in-law not present | N with Mother-in-law not present | N with Mother-in-law present |
| --- | --- | --- | --- | --- | --- | --- | --- | --- |
| Indicator | T(SAAO) | T(APK) | T(SAAO) | T(APK) | T(SAAO) | T(APK) | T(SAAO) | T(APK) |
|  |  |  |  |  | P-value | P-value |  |  |
| *WOMEN* |  |  |  |  |  |  |  |  |
| I make important contributions to my family | 0.06 | 0.06 | 0.02 | -0.01 | 0.56 | 0.33 | 1,299 | 438 |
|  | (0.03) | (0.04) | (0.06) | (0.05) |  |  |  |  |
| Some adults in my family do not make important contributions to the family | -0.09 | -0.11 | -0.05 | -0.23 | 0.78 | 0.51 | 1,299 | 438 |
|  | (0.10) | (0.10) | (0.17) | (0.19) |  |  |  |  |
| I make important contributions to my community | 0.15** | 0.15** | 0.05 | 0.11 | 0.49 | 0.80 | 1,299 | 438 |
|  | (0.07) | (0.07) | (0.12) | (0.15) |  |  |  |  |
| I sometimes refrain from voicing my opinion because I fear being ignored/ridiculed | 0.02 | -0.17** | 0.17* | -0.03 | 0.24 | 0.41 | 1,299 | 438 |
|  | (0.07) | (0.08) | (0.10) | (0.17) |  |  |  |  |
| I have a hard time saying positive things about myself | 0.02 | -0.12 | 0.01 | -0.08 | 0.95 | 0.84 | 1,299 | 438 |
|  | (0.08) | (0.11) | (0.15) | (0.15) |  |  |  |  |
| Women should stand up for themselves to get what they want | 0.11** | 0.09* | -0.05 | -0.09 | 0.05** | 0.02** | 1,299 | 438 |
|  | (0.04) | (0.05) | (0.07) | (0.06) |  |  |  |  |
| Women are usually very busy with work that benefits the household | -0.07 | -0.01 | 0.12** | 0.04 | 0.01*** | 0.40 | 1,299 | 438 |
|  | (0.05) | (0.04) | (0.06) | (0.07) |  |  |  |  |
| Husbands should help wives with household chores like cooking and taking care of children | 0.00 | 0.03 | 0.19** | 0.08 | 0.03** | 0.52 | 1,299 | 438 |
|  | (0.05) | (0.05) | (0.08) | (0.07) |  |  |  |  |
| We can change culture/tradition regarding what men/women do and how they relate | 0.04 | -0.02 | 0.21 | -0.01 | 0.20 | 0.97 | 1,299 | 438 |
|  | (0.07) | (0.07) | (0.13) | (0.14) |  |  |  |  |
| *MEN* |  |  |  |  |  |  |  |  |
| I make important contributions to my family | 0.11*** | 0.04 | -0.04 | 0.01 | 0.03** | 0.65 | 1,299 | 438 |
|  | (0.04) | (0.04) | (0.06) | (0.06) |  |  |  |  |
| Some adults in my family do not make important contributions to the family | -0.15 | 0.02 | 0.02 | -0.14 | 0.29 | 0.40 | 1,299 | 438 |
|  | (0.12) | (0.10) | (0.13) | (0.14) |  |  |  |  |
| I make important contributions to my community | 0.05 | -0.07 | 0.33*** | 0.08 | 0.03** | 0.30 | 1,299 | 438 |
|  | (0.07) | (0.07) | (0.10) | (0.12) |  |  |  |  |
| I sometimes refrain from voicing my opinion because I fear being ignored/ridiculed | -0.07 | -0.05 | 0.14 | 0.08 | 0.17 | 0.42 | 1,299 | 438 |
|  | (0.09) | (0.08) | (0.13) | (0.13) |  |  |  |  |
| I have a hard time saying positive things about myself | 0.02 | -0.04 | -0.03 | -0.04 | 0.67 | 0.98 | 1,299 | 438 |
|  | (0.08) | (0.07) | (0.13) | (0.14) |  |  |  |  |
| Women should stand up for themselves to get what they want | -0.03 | -0.08 | 0.09 | -0.04 | 0.10* | 0.67 | 1,299 | 438 |
|  | (0.05) | (0.06) | (0.08) | (0.10) |  |  |  |  |
| Women are usually very busy with work that benefits the household | 0.04 | -0.04 | 0.03 | -0.08 | 0.95 | 0.64 | 1,299 | 438 |
|  | (0.05) | (0.06) | (0.07) | (0.07) |  |  |  |  |
| Husbands should help wives with household chores like cooking and taking care of children | 0.14*** | 0.03 | -0.13 | -0.13 | 0.01*** | 0.16 | 1,299 | 438 |
|  | (0.05) | (0.05) | (0.08) | (0.09) |  |  |  |  |
| We can change culture/tradition regarding what men/women do and how they relate | 0.19** | -0.01 | 0.14 | 0.22* | 0.75 | 0.09* | 1,299 | 438 |
|  | (0.08) | (0.08) | (0.14) | (0.12) |  |  |  |  |

Note: See Table S10.
